# Lineage-Specific Variation in IR Boundary Shift Events, Inversions, and Substitution Rates among Caprifoliaceae *s.l.* (Dipsacales) Plastomes

**DOI:** 10.3390/ijms221910485

**Published:** 2021-09-28

**Authors:** Seongjun Park, Minji Jun, Sunmi Park, SeonJoo Park

**Affiliations:** 1 Institute of Natural Science, Yeungnam University, Gyeongsan 38541, Gyeongbuk, Korea; seongjun.og@gmail.com; 2 Department of Life Sciences, Yeungnam University, Gyeongsan 38541, Gyeongbuk, Korea; alswi9180@naver.com (M.J.); psm1128@yu.ac.kr (S.P.)

**Keywords:** *accD*, amino acid repeat motifs, *clpP*, intron loss, positive selection, intracellular gene transfer

## Abstract

Caprifoliaceae *s.l*. plastid genomes (plastomes) show that one inversion and two inverted repeat boundary shifts occurred in the common ancestor of this family, after which the plastomes are generally conserved. This study reports plastome sequences of five additional species, *Fedia cornucopiae*, *Valeriana fauriei,* and *Valerianella locusta* from the subfamily Valerianoideae, as well as *Dipsacus japonicus* and *Scabiosa comosa* from the subfamily Dipsacoideae. Combined with the published plastomes, these plastomes provide new insights into the structural evolution of plastomes within the family. Moreover, the three plastomes from the subfamily Valerianoideae exhibited accelerated nucleotide substitution rates, particularly at synonymous sites, across the family. The patterns of *accD* sequence divergence in the family are dynamic with structural changes, including interruption of the conserved domain and increases in nonsynonymous substitution rates. In particular, the *Valeriana* *accD* gene harbors a large insertion of amino acid repeat (AAR) motifs, and intraspecific polymorphism with a variable number of AARs in the *Valeriana accD* gene was detected. We found a correlation between intron losses and increased ratios of nonsynonymous to synonymous substitution rates in the *clpP* gene with intensified positive selection. In addition, two Dipsacoideae plastomes revealed the loss of the plastid-encoded *rps15*, and a potential functional gene transfer to the nucleus was confirmed.

## 1. Introduction

The plastid genome (plastome) of angiosperms is generally conserved and has a quadripartite structure with a pair of inverted repeats (IR) separated by large and small single copy (LSC and SSC) regions [1]. Plastomes generally range from 120 to 160 kb in length and contain 113 unique genes, 79 protein-coding genes, 30 tRNAs, and 4 rRNAs. However, accumulating data on complete plastome sequences exhibit variations in gene and intron content [2]. Plastid gene loss requires functional transfer to the nucleus before the loss in its plastome [3]. Extensive genome rearrangements, including IR boundary shifts, have also been reported for several lineages such as Campanulaceae [4], Caryophyllaceae [5], Fabaceae [6], Geraniaceae [7], Oleaceae [8], and Papaveraceae [9,10].

Lineage-specific variation in the rate of plastome sequence evolution has been documented in angiosperms [5,11,12,13]. Higher substitution rates are associated with structural rearrangements [5,14]. Accelerated rates of nucleotide substitution are affected by the whole genome as well as a subset of protein-coding genes. Compared with other plastid-encoded genes, acetyl-CoA carboxylase subunit β (*accD)*, ATP-dependent Clp protease proteolytic subunit (*clpP*), DNA-directed RNA polymerase subunit α (*rpoA*), some subunits of ribosomal proteins, and the chloroplast factors *ycf1*, *ycf2*, and *ycf4*, show dynamic acceleration [5,9,11,15,16,17,18]. Multiple mechanisms, including dysfunction of DNA replication, repair, and recombination (DNA-RRR) machinery, localized hypermutation, mutagenic retroprocessing, and pseudogenization, have been hypothesized to explain this acceleration [11,14,16,19].

The most extreme accelerations are found in the plastid-encoded *accD* and *clpP*, which have undergone a history of insertions and deletions (indels) of amino acid sequences or intron losses in multiple independent lineages, respectively [9,15]. Several angiosperm lineages have experienced losses in the *accD* or *clpP* genes [10,20,21]. The plastid-encoded *accD* has been functionally replaced via gene transfer to the nucleus or gene substitution in eukaryotic ACCase [15,16,20,22,23,24,25,26]. However, clear evidence of plastid *clpP* transfer to the nucleus in angiosperms is lacking. Highly divergent *accD* and *clpP* genes are likely caused by compensatory mutations in the interactions between nuclear-encoded plastid-targeted subunits [27,28]. This is because the function of the two genes depends on nuclear-encoded proteins that assemble plastid-localized subunits. For example, the plastid-encoded *accD* plays an essential role in the fatty acid biosynthesis pathway [29], which is a subunit of the prokaryotic acetyl–CoA carboxylase (ACC) complex [30]. The prokaryotic ACC complex consists of four subunits, three of which are nuclear-encoded proteins: acetyl–CoA carboxylase subunit α (*ACCA*), biotin carboxyl carrier protein subunit (*ACCB*), and biotin carboxylase subunit (*ACCC*). Plastid-encoded *clpP* is a subunit of the caseinolytic protease (CLP) complex [31] and is involved in multiple processes of chloroplast development [32]. Multiple subunits (CLPP2 to CLPP6) of the CLP complex are encoded in the nucleus [31]. Thus, the nuclear-encoded subunits interact with plastid-encoded subunits.

The honeysuckle family (Caprifoliaceae *sensu lato* [*s.l.*]) comprises approximately 825 species in 28-42 genera with widespread cosmopolitan distribution [33]. Caprifoliaceae *s.l.* has been classified into seven major groups (six subfamilies and one genus): Diervilloideae, Caprifolioideae, Linnaeoideae, Morinoideae, Dipsacoideae, Valerianoideae, and *Zabelia* (Rehder) Makino [34]. A recent phylogenomic study based on nuclear loci and plastome sequences suggested that *Zabelia* is recognized as a new subfamily of Zabelioideae [35]. To date, complete plastomes of 22 genera have been sequenced (National Center for Biotechnology Information; NCBI, accessed on 18 August 2021). The sequenced Caprifoliaceae *s.l.* plastomes range in size from 151.3 to 161.6 kb with a quadripartite organization. Variations in inverted repeat (IR) boundary shifts and nucleotide substitution rates have been documented in the Caprifoliaceae *s.l.* plastomes [34,36]. Despite broader sampling and sequencing across the family, very little is known about the gene evolution, including structural changes and rate variation in the plastid-encoded *accD* and *clpP* genes.

In this study, we generated the complete plastome sequences of three species from the subfamily Valerianoideae and two species from the subfamily Dipsacoideae. Genome organization and nucleotide substitution rates were estimated and compared to the published Caprifoliaceae *s.l.* plastomes. In particular, the correlation between structural evolution and nucleotide substitution rates in the plastid-encoded *accD* and *clpP* genes across this family was examined. In addition, we examined intra- and infraspecific length variations in the *accD* coding region of *Valeriana fauriei* Briq. and *V*. *sambucifolia* f. *dageletiana* (Nakai ex F.Maek.) Hara.

## 2. Results

### 2.1. Plastome Organization

We sequenced and assembled the complete plastomes of three Valerianoideae (*Fedia cornucopiae* (L.) Gaertn., *V. fauriei*, and *Valerianella locusta* (L.) Laterr.) and two Dipsacoideae (*Dipsacus japonicus* Miq. and *Scabiosa comosa* Fisch. ex Roem. & Schult.) species (Figure S1, see Supplementary Materials). Among the five species, the plastome size ranged from 149,809 bp (*V. locusta*) to 160,243 bp (*D. japonicus*) (Table 1). *Dipsacus japonicus* had the largest LSC (87,066 bp), whereas *F. cornucopiae* had the smallest SSC (15,862 bp). The GC content of the *D. japonicus* (38.8%) was higher than that of the other species (Table 1). The three Valerianoideae plastomes encoded 79 protein-coding genes, 30 tRNA genes, and 4 rRNA genes (Table 1). However, the ribosomal protein subunit S15 (*rps15*) appears to be a pseudogene in two Dipsacoideae plastomes (Table 1). Functional replacement by gene transfer of *rps15* from plastid to the nucleus was detected in *Dipsacus* transcriptome data (Figure S2). The plastome of *D. japonicus* was missing *trnT-GGU* and contained two *trnE-UUC* with 90.4% nucleotide identity (Figure S3). The duplicated gene content in the IR region varied as a result of IR expansion and contraction. The intron content also varied owing to the loss of intron in the *clpP* gene. The plastome of *F. cornucopiae* was missing the first intron of the *clpP* gene. The plastome of *V. locusta* was missing both introns in the *clpP* gene (Figure S1).

Among the analyzed Caprifoliaceae *s.l.* plastomes, the largest number of repeat pairs (95) was found in *Morina*, and the fewest repeats (11) were observed in *Valerianella* (Table S1). The average value of repeat pairs from Caprifoliaceae *s.l.* plastomes was ~10 times higher than that from the outgroups (Table S1). The number of repeat pairs from the subfamily Valerianoideae was 2–4 times higher than the average value of repeat pairs from the outgroups, but the number of the repeat pairs from *Valerianella* was similar to that from the outgroups.

**Figure 1:**
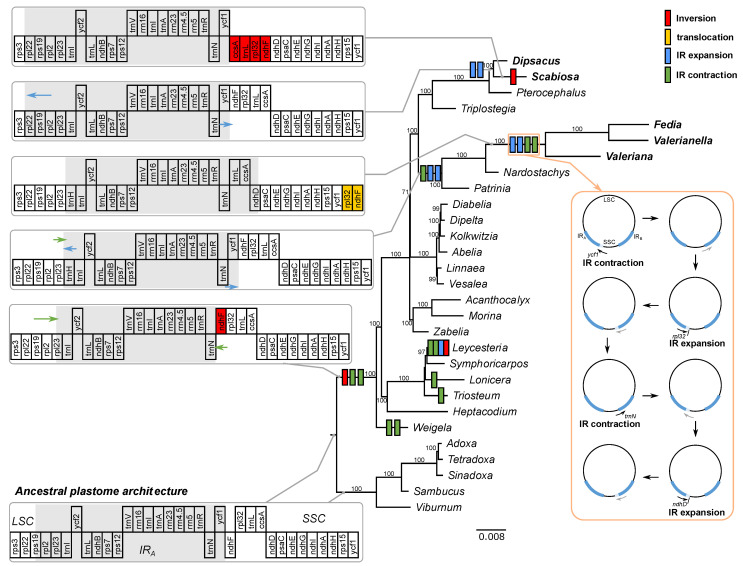
Plastome rearrangement in the analyzed Caprifoliaceae s.l. Schematic diagrams (gray open boxes) of the genomic regions surrounding the inverted repeat (IR) region. Genes drawn below the horizontal line indicate sequences found in an inverted orientation. Gray shadings indicate the IR_A_ region. The hypothetical models for IR expansion and contraction in the common ancestor of Fedia, Valerianella, and Valeriana are illustrated (orange boxes).

To understand the evolutionary history of genome rearrangement in the family, we constructed a phylogenetic tree using 72 plastid genes (Figure 1). The inversions mainly occurred within the IR and SSC regions and some inversion is likely the result of a series of IR expansions and contractions (Figure 1). Based on the most parsimonious interpretation, the ancestral plastome of Caprifoliaceae *s.l.* had three structural changes: (1) an inversion associated with the *ndhF* gene, (2) a contraction at the IR_B_/SSC boundary to *trnN* resulting in the entire *ycf1* gene into the SSC region, and (3) a contraction at the IR_A_/LSC boundary, from the *rps19* to *rpl23* gene (Figure 1). The plastome rearrangement model suggests that lineage- or species-specific events occurred independently after the ancestral structural changes (Figure 1). For example, an independent contraction to *trnI* at the IR_A_/LSC boundary and contraction to *trnR* at IR_A_/SSC occurred in early diverging *Weigela*. Mauve alignment among the subfamily Caprifolioideae identified seven locally collinear blocks (LCBs) with two inversions involving eight breakpoints (Figure S4). Within the subfamily Caprifolioideae, a contraction to *ycf2* at IR_A_/LSC boundary in *Leycesteria* and a contraction to *trnR* at IR_A_/SSC occurred in *Triosteum*, respectively. The inversion in *Leycesteria* is the result of IR expansion at the IR_B_/SSC boundary to *ndhH* and IR contraction at the IR_A_/SSC boundary to *trnN*, resulting in the relocation of the *ycf1-rps15* region (Figure 1). Mauve alignment among the subfamilies Valerianoideae and Dipsacoideae identified five LCBs with six inversions involving seven breakpoints (Figure S4). Contraction to *trnI* at the IR_A_/LSC boundary, followed by an expansion to *trnH* at the IR_B_/LSC boundary and an expansion back to *ycf1*, indicated synapomorphic events in the subfamily Valerianoideae (Figure 1). After that, four IR boundary shift events occurred in the common ancestor of the *Fedia*/*Valerianella*/*Valeriana* clade. The *rpl32-ndhF* region in *Fedia*, *Valerianella*, and *Valeriana* plastomes were relocated as a result of two expansions and two contractions. Expansion at the IR_A_/SSC boundary resulted in the duplication of *ccsA* and included a C-terminal portion of *ndhD* (*Fedia*: 1265 bp; *Valerianella*: 1286 bp; *Valeriana*: 1314 bp), generating a truncated *ndhD* fragment in IR_B_ (Figure 1). Within the subfamily Dipsacoideae, an expansion from *trnH* to *rps3* at the IR_A_/SSC boundary and an expansion from *trnN* to *ycf1* occurred in the common ancestor of the *Dipsacus*/*Scabiosa* clade. The inversion event associated with *ccsA-trnL-rpl32-ndhF* is unique to *Scabiosa* (Figure 1). In *Weigela*, two IR contractions occurred at the IR_A_/LSC and IR_A_/SSC boundaries.

### 2.2. Elevated Substitution Rates in the Plastomes of the Subfamily Valerianoideae

**Figure 2:**
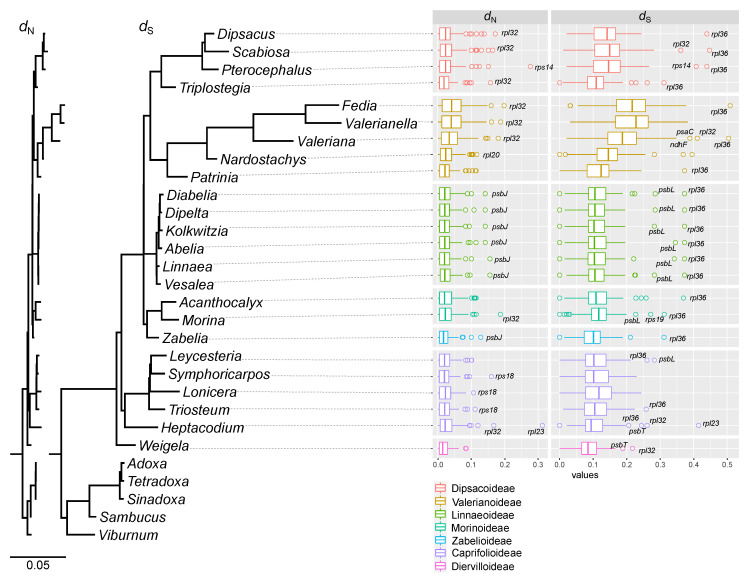
Plastid sequence divergence among the selected Caprifoliaceae *s.l.* Plastid phylograms of nonsynonymous (*d*_N_) and synonymous (*d*_S_) substitution rates based on 72 plastid genes. Boxplots of the values of *d*_N_ and *d*_S_ for individual genes. The box represents values between quartiles, solid lines extend to the minimum and maximum values, outliers are shown as circles and vertical lines in boxes show median values.

The 24 Caprifoliaceae *s.l.* genera and five Adoxaceae genera shared 72 plastid-encoded genes. We excluded seven protein genes from the concatenated data set and substitution rate analysis because they appeared to be pseudogenes or losses (*clpP*, *rps3*, *rps15*, *ycf1*, and *ycf2*) and divergent (*accD* and *ycf3*) among some species within the family. To examine rate variation in the selected plastid genes among the 24 genera, nonsynonymous (*d*_N_) and synonymous (*d*_S_) substitution rates were estimated using the phylogenetic tree as a constraint tree (Figure 2). The *d*_N_ and *d*_S_ values in pairwise comparisons between *Viburnum* and Caprifoliaceae *s.l.* showed that *Fedia*, *Valerianella*, and *Valeriana* from the subfamily Valerianoideae had significantly higher *d*_S_ rates than the other analyzed species (Wilcoxon rank-sum test, *p* < 0.001, after Bonferroni correction; Table S1), except for the comparison between *Valeriana* and *Pterocephalus*. *Narodostachys* and *Patrinia* had only significantly higher *d*_S_ rates than *Weigela* (Wilcoxon rank-sum test, *p* < 0.05, after Bonferroni correction; Table S2). In the case of *d*_N_ rates, only 11 comparisons between *Fedia* and six species (*Kolkwitzia, Symphoricarpos, Triosteum, Triplostegia, Weigela*, and *Zabelia*), between *Valerianella* and four species (*Symphoricarpos, Triplostegia, Weigela*, and *Zabelia*), and between *Valeriana* and *Weigela* showed that *Fedia*, *Valerianella*, and *Valeriana* had significantly higher rates than the other analyzed species (Wilcoxon rank-sum test, *p* < 0.05, after Bonferroni correction; Table S2).

In addition, multiple genes exhibit *d*_N_/*d*_S_ ratios that are greater than one, but likelihood ratio tests (LRTs) with Bonferroni correction indicated that *d*_N_/*d*_S_ for *infA* in *Acanthocalyx*, *ndhD* in Morinoideae/Linnaeoideae/Valerianoideae/Dipscacoideae/Zabelioideae, *psbM* in *Vesalea*, *rbcL* in *Weigela*, *rpoC1* in *Valeriana*, *rpl22* in *Adoxa/Tetradoxa/Sinadoxa*, *rpl32* in *Heptacodium*, *rps2* in *Acanthocalyx, rps4* in *Scabiosa*, *rps14* in *Pterocephalus*, *rps16* in *Dipsacus/Scabiosa/Pterocephalus*, *rps18* in *Abelia*, and *rps19* in *Acanthocalyx* were significantly different (Table S3).

### 2.3. Structural Evolution of Plastid-Encoded accD Gene in Caprifoliaceae s.l. Plastomes

**Figure 3:**
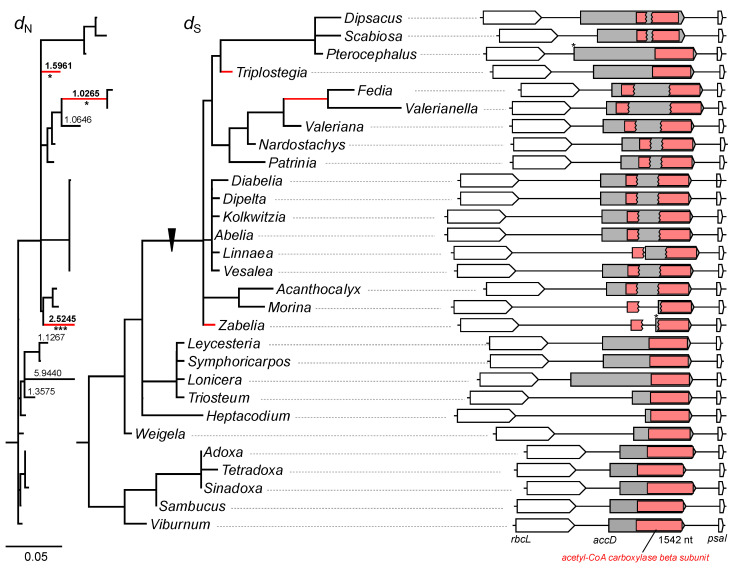
Rapid structural evolution of the *accD* gene. Phylograms show nonsynonymous (*d*_N_) and synonymous (*d*_S_) substitution rates for the *accD* among the analyzed Caprifoliaceae *s.l.* with outgroups. Scale bar indicates the number of substitutions per site. Gray boxes indicate the predicted open reading frames (ORFs). Red rectangles indicate the conserved domain in the ORFs. Asterisks indicate that the ORF has an alternative start codon. Branch lengths are drawn to the same scale based on *d*_N_ and *d*_S_ substitutions per site. Branches with significantly higher *d*_N_/*d*_S_ ratios, as determined by likelihood ratio test are marked with asterisks (*, *p* < 0.05; ***, *p* < 0.001 after Bonferroni correction).

The length of the acetyl–CoA carboxylase beta subunit D (*accD*) open reading frame (ORF) varied remarkably among the examined Caprifoliaceae *s.l.* plastomes, ranging from 711 bp in *Heptacodium* to 2517 bp in *Lonicera* (Figure 3). The comparison revealed two histories of insertion events within the N- and C-terminal regions, resulting in the expansion or truncation of the *accD* ORF. The most parsimonious interpretation is that the *accD* genes were interrupted by the insertion of amino acids in the common ancestor of Caprifoliaceae *s.l.* (Figure 3). The second insertion of amino acids occurred in the common ancestor of *Zabelia*, Morinoideae, Linnaeoideae, Valerianoideae, and Dipscacoideae (except *Pterocephalus* and *Triplostegia)*, which was split into two portions of the conserved domain (Figure 3 and Figure S4).

In particular, the *accD* ORFs of *Morina*, *Linnaea*, and *Zabelia* contain a part of the conserved domain, but the catalytic sites are included (Figure S2). Conserved domain (CD) searches identified a portion of Apolipoprotein, MSCRAMM_ClfB, MSCRAMM_SdrC, GAT1, PPK08581, PPK05901, SMC_N, pneumo_PspA, and rplD subfamilies surrounding the conserved domain of *accD* in the predicted ORF (Table S4). The presence of MSCRAMM_ClfB in *Symphoricarpos*, Linnaeoideae, and Adoxaceae indicated that this event occurred in the most recent common ancestor of each clade (Table S4). Protein sequence alignment of all inserted regions from the *accD* was highly divergent with low amino acid identities of 2.9–96.7% (Figure S4). However, two lineages—*Dipsacus/Scabiosa* and Linnaeoideae—had high amino acid identities of 94.3% and 83.1–96.7%, respectively.

The *d*_N_ and *d*_S_ were calculated for the selected Caprifoliaceae *s.l.* and outgroup using only the conserved domain sequences to test the effect of the insertion on nucleotide substitution rates of the *accD* gene. The *d*_N_ values for *accD* in the selected Caprifoliaceae *s.l.* were significantly higher than in the outgroup (Wilcoxon rank-sum test, *p* < 0.05; Figure S3). Seven branches with *d*_N_/*d*_S_ ratios > 1 were detected, but LTRs showed that three branches, *Fedia/Valerianella*, *Triplostegia*, and *Zabelia,* were significantly different (*p* < 0.00001 after Bonferroni correction, Figure 3). The RELAX analysis indicated that the *accD* experienced significantly intensified selection in the *Zabelia*, Morinoideae, Linnaeoideae, Valerianoideae, and Dipsacoideae clade (*k* = 1.96, *p* = 0.020, likelihood ratio [LR] = 5.38), and the Caprifoliaceae *s.l.* clade (*k* = 2.25, *p* = 0.032, LR = 4.60).

### 2.4. Length Variation in the Valeriana accD Gene

**Figure 4:**
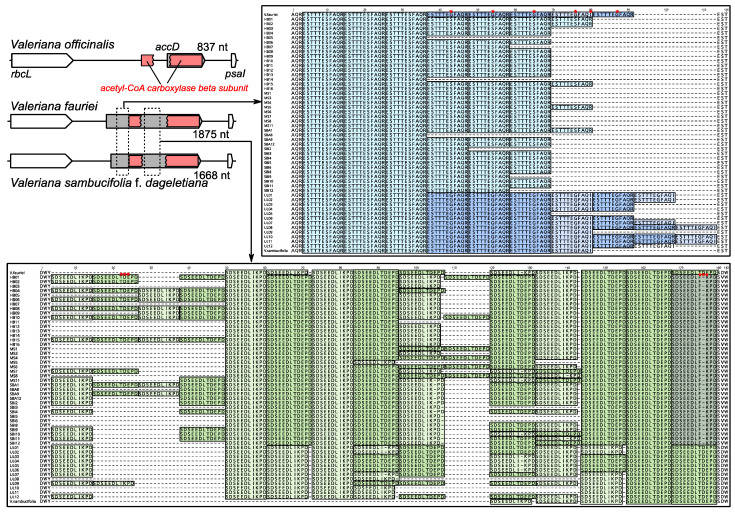
Length variation in the plastid *accD* of *Valeriana*. Schematic diagram of the genomic regions surrounding the *accD* from three *Valeriana* species. Gray boxes indicate the predicted open reading frames (ORFs). Red rectangles indicate the conserved domain in the ORFs. Dashed boxes indicate two hotspot regions in the *accD* gene. Each amino acid sequence of the two hotspot regions of the *accD* copies from 52 *Valeriana* individuals. Blue and green boxes indicate amino acid repeat (AAR) motifs. Asterisks indicate an amino acid sequence mismatch of the AARs (ESTTTESFAQR and SDSEEDLIKPD), respectively. *V. fauriei*: HB, MS, SBA, and SBI; *V. sambucifolia* f. *dageletiana*: UL.

*Valeriana fauriei* plastome contains an expanded *accD* gene, which is interrupted by amino acid repeats (AARs) surrounding the conserved domains. Compared with the other available *Valeriana* plastomes, *V. officinalis* contains a truncated *accD* gene in its genome, whereas *V. sambucifolia* f. *dageletiana* contains an expanded *accD* like *V. fauriei accD* (Figure 4A). To evaluate the variability of the AAR motifs in the *accD* gene of *V. fauriei* and *V. sambucifolia* f. *dageletiana,* we designed a PCR primer that targets two hotspot regions of *accD* (Figure 4A). The amplicon sizes of the region ranged from 966 bp to 1239 bp (Table S5). Alignment of the two region sequences of the 50 individuals with two *accD* sequences from two plastomes revealed intra-and infraspecific variation of the *accD* in *Valeriana* (Figure 4). Two hotspot regions show length variation, consisting of (1) 3 to 10 repeats of “ESTTTESFAQR” and (2) 5 to 14 repeats of “SDSEEDLIKPD”, although there are one or three different amino acid sequences (Figure 4).

### 2.5. Correlation between Structural Change and Substitution Rates in the Plastid-Encoded clpP Gene

**Figure 5:**
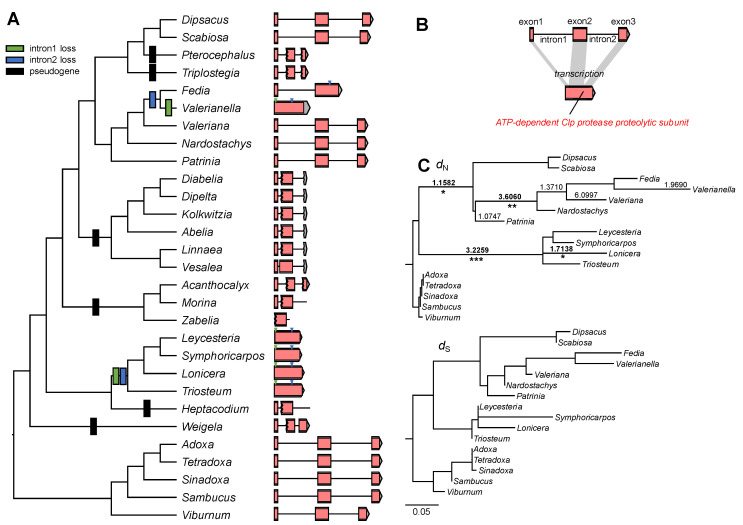
Structural evolution of the *clpP* gene: (**A**) phylogenetic distribution of pseudogene or intron loss among the selected Caprifoliaceae *s.l.* and five outgroups. Arrowheads indicate the positions of the first (green) and second (blue) intron; (**B**) schematic diagram of the structure of *clpP*. Pink boxes indicate the conserved domain of caseinolytic protease; (**C**) phylograms showing nonsynonymous (*d*_N_) and synonymous (*d*_S_) substitution rates for the *clpP* genes among 16 species that have intact gene sequences. Branch lengths are drawn to the same scale based on *d*_N_ and *d*_S_ substitutions per site. Branches with significantly higher *d*_N_/*d*_S_ ratios determined by likelihood ratio test are marked with asterisks (*, *p* < 0.05; **, *p* < 0.01; ***, *p* < 0.001 after Bonferroni correction).

The phylogenetic distribution of *clpP* content in the selected Caprifoliaceae plastomes showed that this gene had been pseudogenized multiple times in the family (Figure 5A). BlastN searches using the plastid-encoded *clpP* from *Viburnum* identified partial exons or introns of *clpP* in *Pterocephalus*, *Triplostegia*, *Heptacodium*, *Weigela*, Linnaeoideae, Morinoideae, and Zabeliaoideae, which lacked a conserved domain (Figure 5). Only *Dipsacus, Scabiosa, Fedia, Leycesteria, Nardostachys, Symphoricarpos, Valerianella, Valeriana, Lonicera*, and *Triosteum* plastomes contain *clpP* genes that differ in intron content (Figure 5A,B). Similar to the *Valerianella clpP* gene*,* the *clpP* gene of *Leycesteria, Lonicera, Symphoricarpos,* and *Triosterum* are missing both introns. The phylogenetic distribution indicated that the loss of the second intron occurred in the *Fedia/Valerianella* clade (Figure 5).

To examine the correlation between structural changes and substitution rates, *d*_N_ and *d*_S_ were calculated using the 16 *clpP* gene sequences available (Figure 5C). Seven branches with *d*_N_/*d*_S_ values >1 were detected. However, LRTs revealed that only four branches, *Fedia/Valerianella/Valeriana/Nardostachys*, Valerianoideae, *Lonicera*, and Caprifolioideae, were significantly under positive selection (*p* < 0.00001 after Bonferroni correction). The RELAX analysis indicated that *clpP* experienced significantly intensified selection in the analyzed Caprifoliaceae *s.l.* (*k* = 2.62, *p* = 0.001, LR = 10.96). Additional analyses indicated that the intronless *clpP* gene experienced significantly intensified selection in the subfamily Caprifolioideae (*k* = 3.18, *p* = 0.000, LR = 16.06). In *Fedia* and *Valerianella*, the *clpP* gene showed intensified selection, but this was not significant (*k* = 1.33, *p* = 0.443, LR = 0.59).

## 3. Discussion

Sequencing of the *F. cornucopiae*, *V. fauriei, V. locusta, D. japonicus*, and *S. comosa* plastomes revealed that they are distinct from the published plastomes of Caprifoliaceae *s.l.* These plastomes exhibit dynamic changes in structure, gene and intron content, and lineage-specific rate acceleration. Our results also showed a correlation between mutation rates and structural variation in the *accD* and *clpP* genes across Caprifoliaceae *s.l.* and interruption of the *accD* gene in the genus *Valeriana*. The loss of plastid-encoded *rps15* was observed in *D. japonicus* and *S. comosa* plastomes. Comparative analysis of the gene content among Caprifoliaceae *s.l.* plastomes suggests the *rps15* is lost in the common ancestor of *Dipsacus*, *Scabiosa*, and *Pterocephalus*. Functional replacement of the *rps15* from plastid to the nucleus occurs in these lineages, although we found evidence for a plastid-to-nucleus gene transfer in the *Dipsacus* transcriptome. Additional nuclear transcriptome data for *Scabiosa* and *Pterocephalus* are needed for further investigation.

With the five plastomes, we selected the published plastomes of 19 additional genera from Caprifoliaceae *s.l.* and five genera from Adoxaceae to reconstruct the ancestral plastome in Caprifoliaceae *s.l.* and rearrangement events in each genus. Our results revealed three synapomorphic events (one inversion and two contractions of IR) in the Caprifoliaceae *s.l.* (Figure 1). A previous study showed that the inversion associated with the *ndhF* gene occurred in Adoxaceae [34]. However, compared with angiosperm plastomes, the ancestral Caprifoliaceae *s.l.* plastome has an inversion between *ndhF*. The comparison of the LCBs and IR boundary shift models indicates that independent events occurred in a lineage- or species-specific manner (Figure 1). The model suggests that the IR expansion and contraction were the main mechanisms for changes in gene order in *F. cornucopiae*, *V. fauriei,* and *V. locusta* plastomes. A double-strand break, followed by strand invasion, expansion, and recombination in IR [37], is a potential mechanism for IR expansion in the family. After ancestral inversion, one additional inversion event associated with *ccsA-trnL-rpl32-ndhF* was identified, unique to *Scabiosa*. Dispersed repeats can cause inversions and there is a correlation between the number of repeats and plastome rearrangements [6,38]. However, *Scabiosa* has a small number of repeats in the family, although it has a relatively large number of repeats compared to the outgroups (Table S1). Moreover, dispersed repeat sequences were not found surrounding the inversion block in the *Scabiosa* plastome, which suggests that a different mechanism of inversion may be involved. Several ebb-and-flow expansions and contractions were also observed in the analyzed Caprifoliaceae *s.l*. plastomes.

Lineage-specific variation occurred in the Caprifoliaceae *s.l.* plastomes, showing that *Fedia, Valeriana*, and *Valerianella* had significantly accelerated *d*_S_ in comparison (Figure 2). Structural rearrangements have contributed to higher substitution rates [5,14]. Compared with the analyzed Caprifoliaceae *s.l.* plastomes, the three plastomes showed increased levels of structural divergence (Figure 1). However, IR boundary shifts are the main mechanisms responsible for the genomic changes in this lineage. This variation appears to result from genome-wide acceleration, arguing against localized hypermutation, mutagenic retroprocessing, and pseudogenization [16]. Mutated and changed DNA-RRR machinery could be a potential mechanism to explain these phenomena. In the subfamily Valerianoideae, expanded plastome sequencing and examination of organellar-targeted DNA-RRR genes would be needed to explain the causes and consequences of fast-evolving plastomes in this lineage.

Our analysis showed that interrupted *accD* evolution has occurred repeatedly across Caprifoliaceae *s.l.* (Figure 3). Many plastomes show that the *accD* is unrecognized and unannotated because of its extreme divergence [34,36]. In these cases, the *accD* appears to have been lost and possibly transferred to the nucleus, as intracellular gene transfer (IGT) is an ongoing process in angiosperms [3]. Evidence supports the functional replacement of the *accD* gene by gene transfer to the nucleus or gene substitution of nuclear homologs, including coexistence of the nuclear-encoded, plastid-targeted eukaryotic ACCase, prokaryotic ACCase, and the plastid-encoded *accD* [15,16,20,22,23,24,25,26]. However, previous studies have provided some evidence that highly divergent or truncated *accD* genes may be functional in plastids. For example, the divergent copy of the plastid-encoded *accD* in *Lamprocapnos spectabilis* (L.) Fukuhara (Papaveraceae) is transcribed [9]. A functional replacement of truncated *accD* to the nucleus was found in *Trachelium caeruleum* L. (Campanulaceae) [20], *Hypseocharis bilobata* Killip, and *Monsonia emarginata* (L.f.) L’Hér. (Geraniaceae) [15]. In the selected Caprifoliaceae *s.l.* plastomes, many *accD* reading frames remain intact, and several have truncated with the catalytic sites, which suggests that it probably encodes a functional protein. The intensity of both purifying (most branches are *d*_N_/*d*_S_ < 1) and positive selection (three branches, *Fedia/Valerianella*, *Triplostegia*, and *Zabelia* with *d*_N_/*d*_S_ > 1) also indicated that the *accD* is under selective constraint or adaptive changes. To fully understand the evolution of ACCase among Caprifoliaceae *s.l.* genomes, searching the assembled nuclear transcriptomes are required. In addition, we examined the variability of the AAR motifs in the *accD* gene and found evidence of intraspecific length variation in the *Valeriana*. One possible mechanism for length polymorphism is replication slippage and recombination [39]. Similar patterns of the *accD* gene have been previously shown to have repetitive amino acid sequence motifs in *L. spectabilis* [9] and *Medicago truncatula* Gaertn. [40]. Gurdon and Maliga [40] suggested that repetitive amino acid motifs within the *accD* could be recombinationally driven.

Previous studies have shown that multiple lineages experience independent loss [7,14,41,42,43]. Some cases of losses are because the *clpP* gene is unrecognized and unannotated owing to high divergence including structural changes. For example, the *clpP* gene was annotated as a loss in the five Actinidiaceae plastomes [42,43], but the plastomes contain *clpP*-like ORFs that are missing the two introns with a completely conserved domain. The lack of internal stop codons or frameshifts suggests the functionality of the plastid-encoded *clpP*. The *Geranium* and *Monsonia clpP* genes were annotated as pseudogenes or losses [7,14]; however, extremely divergent and intronless *clpP* ORFs are found in the lineages [15]. *ClpP* is involved in important chloroplast processes [32]. If the divergent ORFs are pseudogenes, functional replacement by gene transfer or gene substitution must occur. However, the evolutionary fate of the plastid-encoded *clpP* loss has not been reported in angiosperms. We identified at least six potential pseudogenizations of *clpP* and independent losses of one or both introns in the analyzed Caprifoliaceae *s.l.* (Figure 5). Using the conserved domain sequence of *clpP* as a query, we did not find any evidence of divergent *clpP*-like ORFs that were intact. Only two lineages contained the *clpP* gene, showing dynamics in intron content. The phylogenetic distribution showed the evolutionary history of intron loss events in the subfamily Valerianoideae, in which the loss of the second intron in the common ancestor of *Fedia* and *Valerianella*, followed by loss of the first intron independently in *Valerianella* (Figure 5). The subfamily Caprifolioideae plastome contains intronless *clpP*, but the history of loss events is unclear from the present data. Direct genomic deletion, exonization of introns, retroprocessing, and gene conversion with foreign copies are possible mechanisms of intron loss [44]. Intensified positive or negative selection in the *clpP* gene among Caprifoliaceae *s.l.* suggests that positive selection may act on the nuclear-encoded plastid-targeted genes. Cytonuclear coevolution between plastid- and nuclear-encoded subunits in Caprifoliaceae *s.l.* should be explored to test this hypothesis.

## 4. Materials and Methods

### 4.1. Genome Sequencing, Assembly, and Annotation

Total genomic DNA (gDNA) from *V. fauriei* and *V. locusta* from the subfamily Vaerianoideae, as well as *D. japonicus*, and *S. comosa* from the subfamily Dipsacoideae (Table S5), were isolated from fresh leaf tissues of a single individual using the Exgene Plant SV Mini Kit (GeneAll, Seoul, South Korea) following the manufacturer’s protocol. The gDNA of *F. cornucopiae* was provided by the Royal Botanic Gardens Kew DNA and Tissue Collection (Table S5). The gDNAs were sequenced using an Illumina Hiseq2500 sequencing platform (Illumina, San Diego, CA, USA), generating 6 Gb of 150 bp paired-end (PE) reads from a 550 bp insert library.

The PE reads were assembled de novo using Velvet v1.2.10 [45] using multiple k-mers (99 to 141). For each plastome, the longest contigs that reflected a complete plastome with only one copy of the IR were aligned manually, and the consensus was taken as the final genome sequence. Finished plastomes were annotated using a BLAST-like algorithm in Geneious Prime 2021.1.1 (www.geneious.com, accessed on 18 August 2021) with the genes of Nicotiana tabacum L. plastome (NC_001879) as the reference, and the open reading frames (ORFs) were confirmed using the “Find ORFs” option. Circular plastome maps were drawn using OrganellarGenomeDRAW (OGDRAW) v1.3.1 (https://chlorobox.mpimp-golm.mpg.de/OGDraw.html, accessed on 18 August 2021) [46]. The plastomes were deposited in GenBank (accession numbers MZ934745-MZ934749).

### 4.2. Comparative Analyses

Repetitive DNA sequences in each plastome were identified by performing “blastn” searches using BLAST + v2.6.0 [47] against itself, with a word size of 11, an e-value of 1 × 10^−6^. The newly sequenced plastomes and the 19 published Caprifoliaceae *s.l.* plastomes were aligned with the outgroup *Viburnum betulifolium* Batalin from Adoxaceae using the “progressiveMauve” algorithm in Mauve v2.3.1 [48] in Geneious Prime. The National Center for Biotechnology Information (NCBI) Conserved Domain Database (CDD) v3.19 was used for functional domain annotation (https://www.ncbi.nlm.nih.gov/Structure/cdd/wrpsb.cgi, accessed on 18 August 2021) [49]. Transcriptome from *Dipsacus asperoides* C.Y.Cheng & T.M.Ai was assembled de novo with Trinity [50] using the Sequence Read Archive (SRA) (SRR2043985). The potential nuclear-encoded transcript was identified in the transcriptome by using “blastn” (e-value cutoff of 1 × 10^−10^) with the plastid-encoded *rps15* gene sequence from *Triplostegia glandulifera* Wall. ex DC. as a query. Chloroplast transit peptide (cTP) was predicted by TargetP v1.1 [51].

### 4.3. Estimation of Substitution Rates

In total, 72 plastid protein-coding genes shared by all selected 29 taxa from newly sequenced plastomes and from the published plastomes were sampled (Table S1). Individual genes were aligned using the back-translation method with MAFFT [52] in Geneious Prime. The constraint tree was generated using the maximum likelihood method in IQ-TREE v2.1.2 [53] with concatenated sequence alignment. To estimate the rates of nucleotide substitution, all genes and the concatenated sequences were analyzed individually. The nonsynonymous (*d*_N_) and synonymous (*d*_S_) substitution rates for datasets were calculated in PAML v4.8 [54] with the constraint tree. Codon frequencies were estimated using the F3 *×* 4 model. LRTs were performed in Hyphy v2.5.23(MP) [55] to test *d*_N_/*d*_S_ changes using the MG94xREV codon model. To test for potential relaxed selection, the RELAX [56] implemented in HyPhy was used on the Datamonkey Adaptive Evolution Server (https://www.datamonkey.org/, accessed on 18 August 2021) [57].

### 4.4. Survey of Variability in the Plastid-Encoded accD Gene

To examine length variation in the *accD* gene at the inter- and intraspecific levels, 38 *V. fauriei* individuals and 12 *V. sambucifolia* f. *dageletiana* individuals were sampled (Table S5). The gDNAs were extracted from the fresh leaves or herbarium specimens using the GeneAll Kit, or the methods described by Allen et al. [58]. Variable regions in the *accD* gene were amplified by PCR using specific primers designed with Primer3 in Geneious Prime (64F: 5*′*-AACTCTTATGATTCGGTTTCTCGT-3*′* and 1328R: 5*′*-ATACCGGTTTGAATAGCCTCAGTT-3*′*). Each reaction was 50 μL in volume, including 38.75 μL of distilled water, 5 μL of 10 × Taq Reaction Buffer, 1 μL of dNTPs (10 mM), 0.25 μL of DiaStar^TM^ Taq polymerase (5 units/μL, Solgent Co., Daejeon, Korea), 1 μL of each primer (10 pmole/μL), and 1 μL of total gDNA (20 ng). All reactions consisted of included an initial denaturation step (95 °C for 2 min), 35 cycles of denaturation (95  °C for 20 s), annealing (60  °C for 40 s), and extension (72  °C for 1 min 30 s), followed by a final extension (72  °C for 5 min). The PCR products were purified using a PCR purification kit (MGmed, Korea) according to the manufacturer’s protocol. Sequencing of PCR products was carried out using an ABI 3730xl DNA Analyzer (Applied Biosystems, Foster City, CA, USA) at Solgent Co. The nucleotide sequences of the plastid *accD* copies were aligned using MUSCLE [59] in Geneious Prime.

## Figures and Tables

**Table 1:** Comparison of Caprifoliaceae plastomes sequenced in this study.

Taxon	Dipsacoideae		Valerianoideae		
	*Dipsacus* *japonicus*	*Scabiosa comosa*	*Fedia* *cornucopiae*	*Valeriana fauriei*	*Valerianella locusta*
Size (bp)	160,243	159,651	152,196	155,302	149,809
LSC length (bp)	87,066	87,477	82,960	85,541	82,103
SSC length (bp)	17,850	18,716	15,862	15,159	15,796
IR length (bp)	27,664	26,729	26,687	27,301	25,955
Number of protein-coding genes	78 (6)	78 (7)	79 (4)	79 (4)	79 (4)
Number of tRNA genes	29 (8)	30 (7)	30 (7)	30 (7)	30 (7)
Number of rRNA genes	4 (4)	4 (4)	4 (4)	4 (4)	4 (4)
Number of introns	21 (5)	20 (5)	21 (5)	21 (5)	19 (5)
GC content (%)	38.8	38.7	38.2	38.4	38.1

## Data Availability

Data are available in a publicly accessible repository. GenBank accession numbers for the new sequences are MZ934745-MZ934749 and MZ954788-MZ954837.

## Supplementary Materials

The following are available online at https://www.mdpi.com/article/10.3390/ijms221910485/s1, Figure S1: Maps for the newly sequenced plastomes, Figure S2: Nucleotide and amino acid sequences of the nuclear-encoded *RPS15* gene from *Dipsacus*, Figure S3: Duplication of the *trnE-UUC* gene in *Dipsacus japonicus* plastome, Figure S4: Structural alignments of Caprifoliaceae *s.l.* plastomes using Mauve. Figure S5: Amino acid sequence alignments of the plastid-encoded *accD* of Caprifoliaceae *s.l.*, Figure S6. Boxplots of the values of nonsynonymous and synonymous substitution rates of the plastid-encoded *accD* for Caprifoliaceae *s.l.* and outgroups, Table S1: GenBank accession numbers for taxa used in this study, Table S2: Pairwise Wilcoxon rank-sum tests of *d*_N_ and *d*_S_ values among plastid genes within Caprifoliaceae *s.l.*, Table S3: Positive selection on Caprifoliaceae *s.l.* plastid genes, Table S4: CD-search results of the plastid-encoded *accD* gene from Caprifoliaceae *s.l.*, Table S5: Material information and GenBank accession numbers for length variation in the plastid-encoded *accD* gene.
