# Supplementary material for: Lineage-Specific Variation in IR Boundary Shift Events, Inversions, and Substitution Rates among Caprifoliaceae s.l. (Dipsacales) Plastomes
Source: Int J Mol Sci. 2021 Sep 28;22(19):10485. doi: 10.3390/ijms221910485 (PMC8508905; doi:10.3390/ijms221910485)
Supplement: Supplementary file 1 [file ijms-22-10485-s001.zip › ijms-1380233-supplementary.pdf]

Article title: Lineage-specific variation in IR boundary shifting events, inversions, and substitution rates, among Caprifoliaceae *s.l.* plastomes

Authors: Seongjun Park, Minji Jun, Sunmi Park, and SeonJoo Park

Supplementary information

Figure S1. Maps for the newly sequenced plastomes.

Figure S2. Nucleotide and amino acid sequences of the nuclear-encoded *RPS15* gene from *Dipsacus*.

Figure S3. Duplication of the *trnE-UU*C gene in *Dipsacus japonica* plastome.

Figure S4. Structural alignments of Caprifoliaceae *s.l.* plastomes using Mauve.

Figure S5. Amino acid sequence alignments of the plastid-encoded *accD* of Caprifoliaceae *s.l.* and outgroups.

Figure S6. Boxplots of the values of nonsynonymous and synonymous substitution rates of the plastid-encoded *accD* for Caprifoliaceae *s.l.* and outgroups.

Table S1. GenBank accession numbers for taxa used in this study.

Table S2. Pairwise Wilcoxon rank sum tests for d_N and d_S values among plastid genes within Caprifoliaceae *s.l.*.

Table S3. Positive selection on Caprifoliaceae *s.l.* plastid genes.

Table S4. CD-search results of plastid-encoded *accD* gene from Caprifoliaceae *s.l.*.

Table S5. Material information and GenBank accession numbers for length variation in the plastid-encoded *accD* gene.

Figure S1. Maps for the newly sequenced plastomes. Thick lines on the inner circle indicate the inverted repeats (IRA and IRB), which separate the genome into small (SSC) and large (LSC) single-copy regions. Genes on the inside and outside of the map are transcribed in clockwise and counterclockwise directions, respectively. The ring of bar graphs on the inner circle indicates the GC content in dark grey. ψ denotes a pseudogene. A) Plastome map for *Dipsacus japonicus* B) Plastome map for *Scabiosa comosa* . C) Plastome map for *Fedia cornucopiae* . D) Plastome map for *Valeriana fauriei* . E) Plastome map for *Valerianella locusta.*

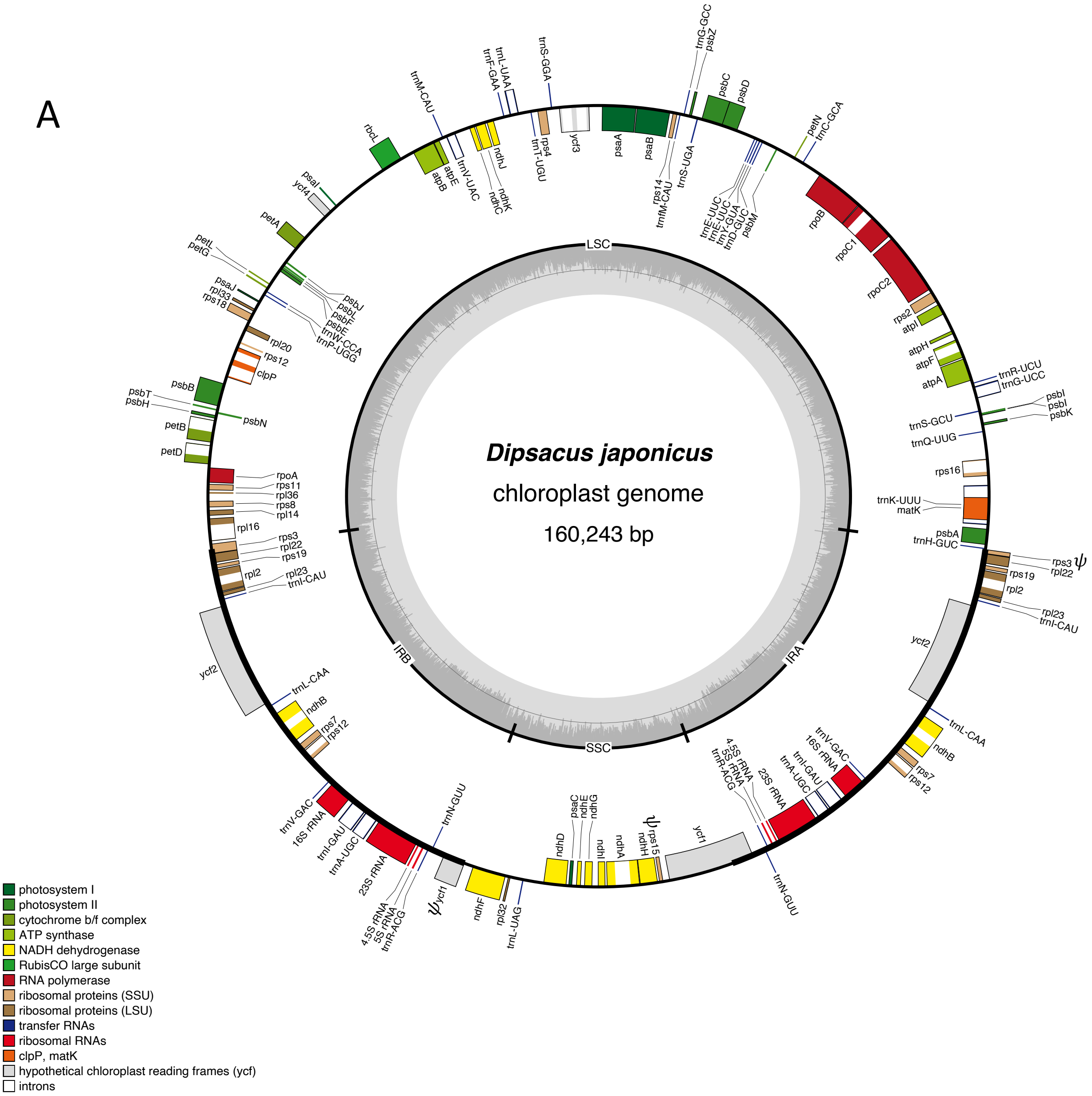

Figure S1. (continued)

B

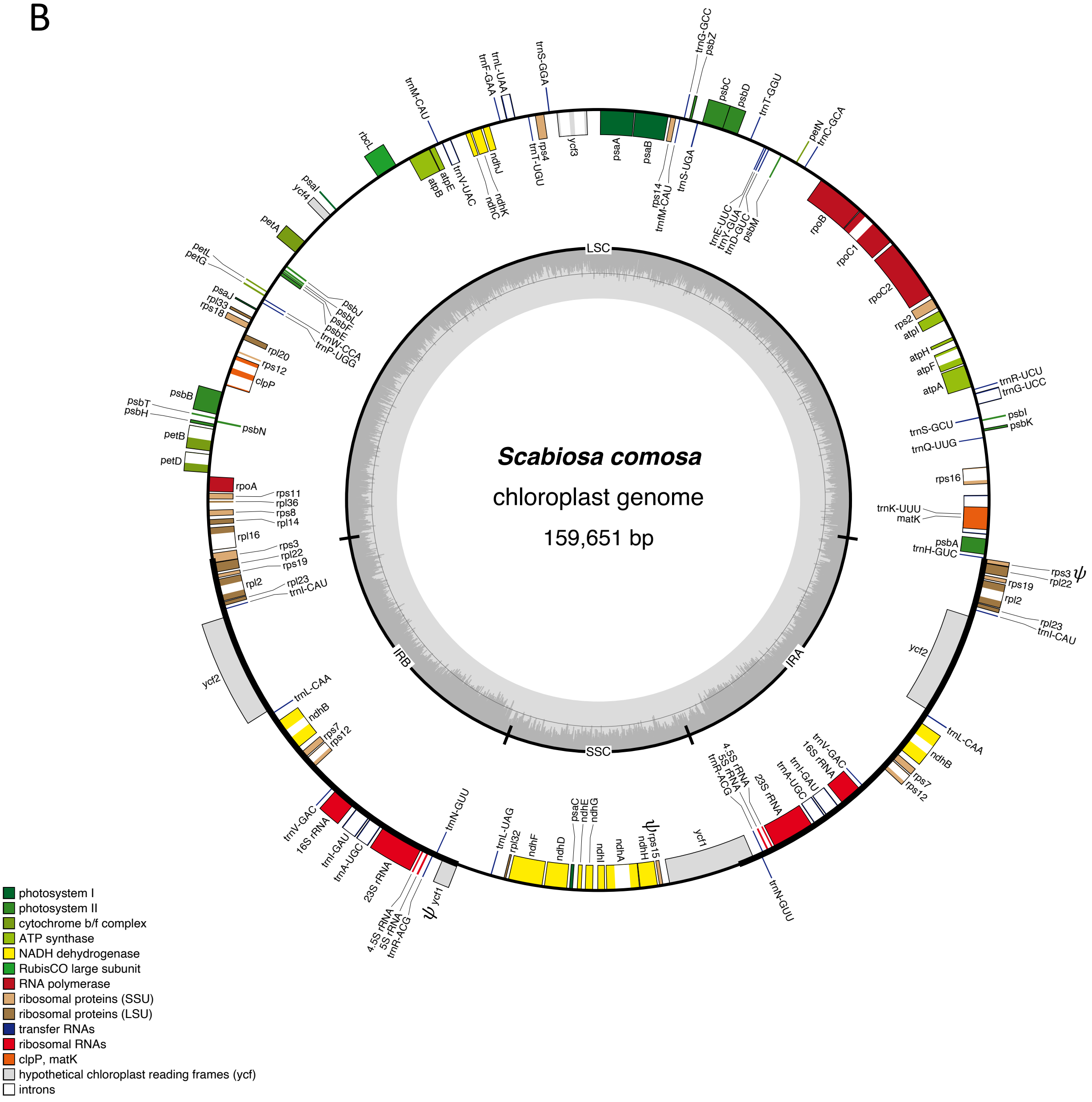

Figure S1. (continued)

C

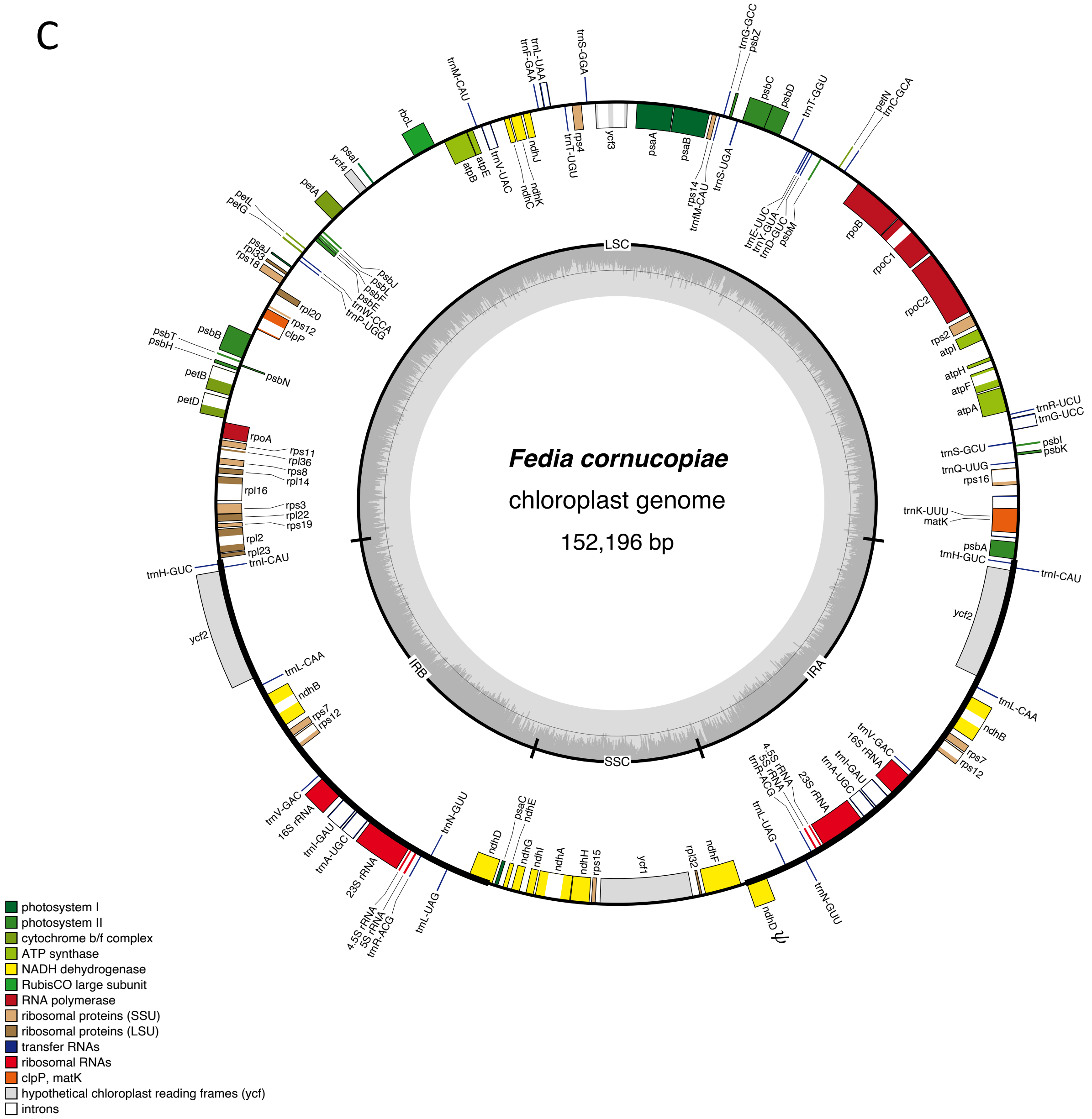

Figure S1. (continued)

D

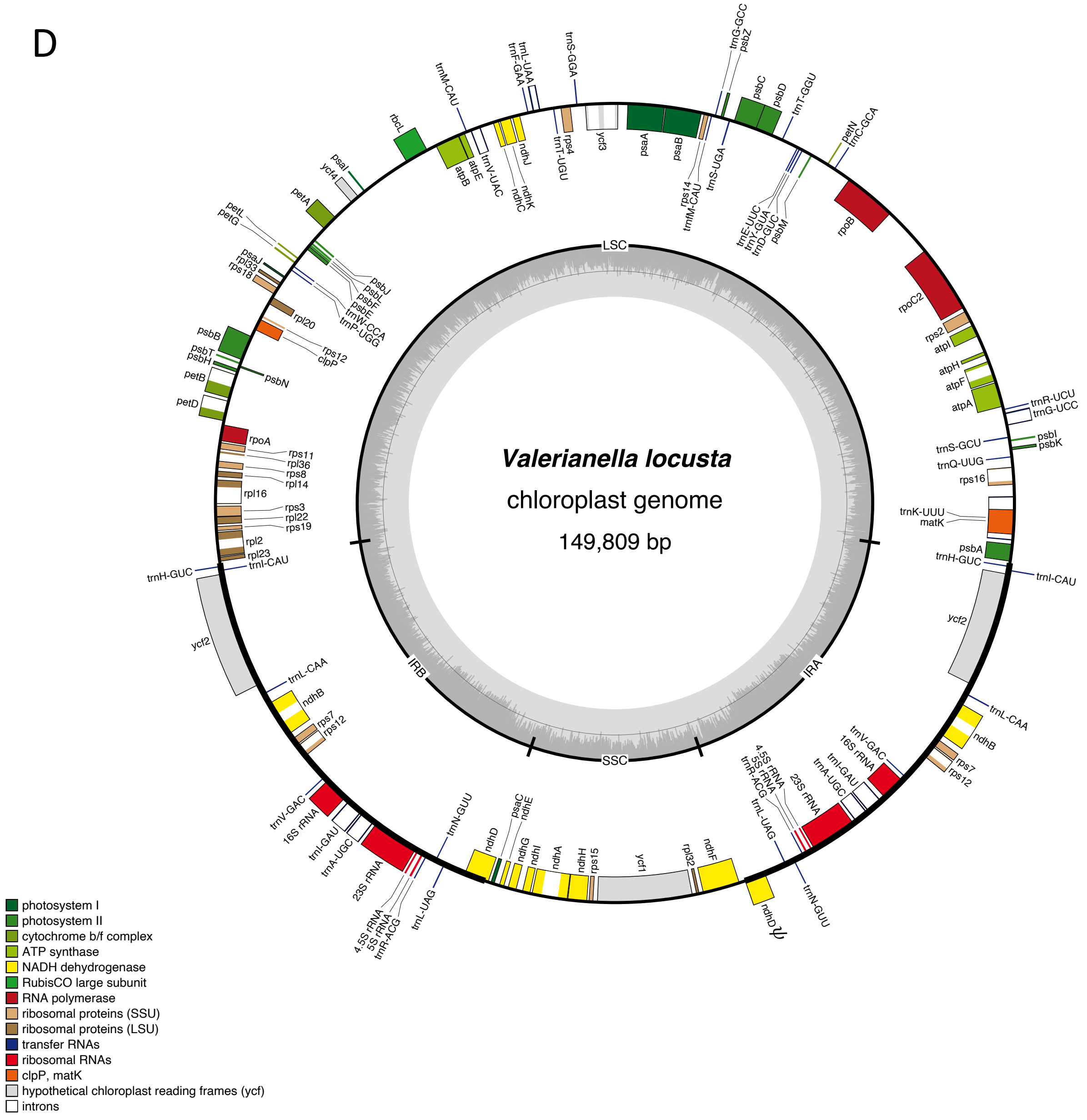

Figure S1. (continued)

E

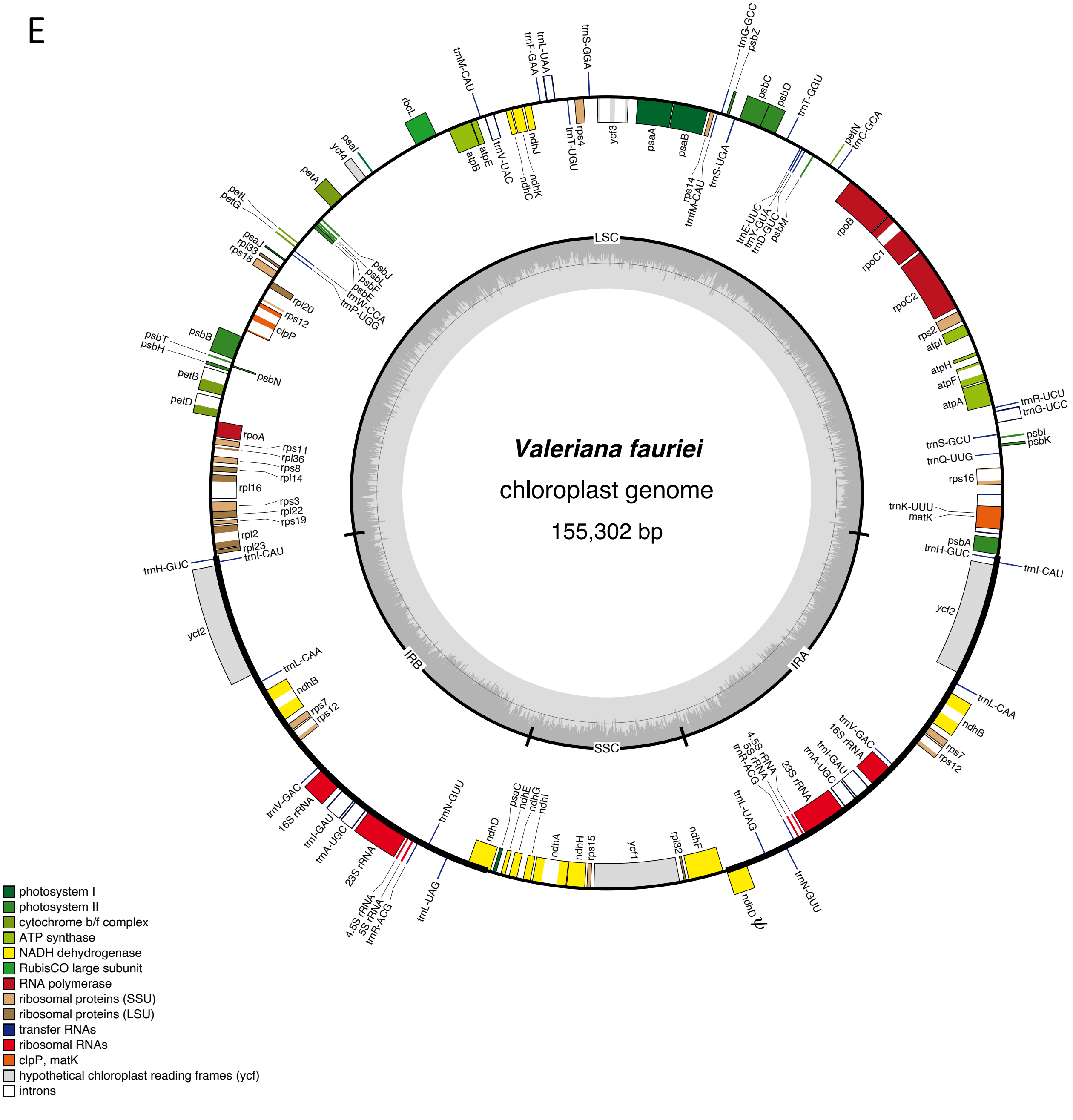

Figure S2. Nucleotide and amin acid sequences of the nuclear-encoded *RPS15* gene from *Dipsacus*. Green and red boxes indicate plastid transit peptides and a conserved domain of ribosomal protein S15, which are predicted using TargetP and CDD, respectively.

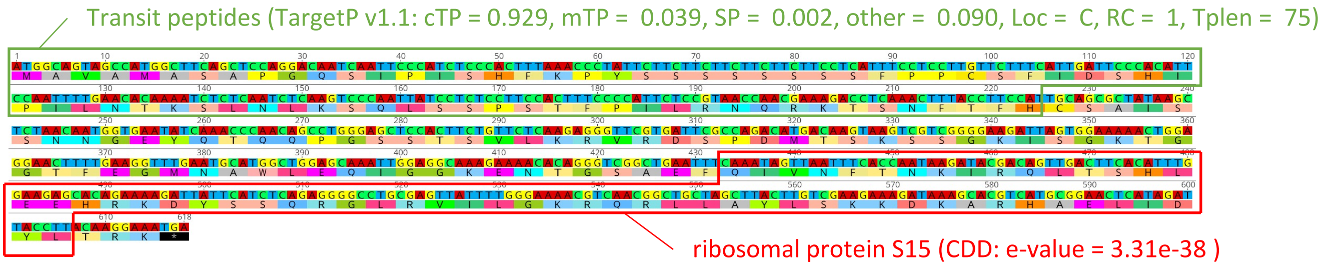

Figure S3. Duplication of the *trnE-UUC* gene in the *Dipsacus japonicus* plastome. (A) Nucleotide alignment of *Dipsacus trnE-UUC* copies with five copies from related species and *Nicotiana tabacum*. The sequence logo indicates the most conserved bases at each position. The numbers "1" and "2" represent paralogs of *trnE-UUC*. (B) Predicted secondary structures for the two *trnE-UUC* sequences of *Dipsacus*. The substitution sites are highlighted with blue and red circles against Nicotiana and Dipsacus1 *trnE-UUC* copies, respeictvely.

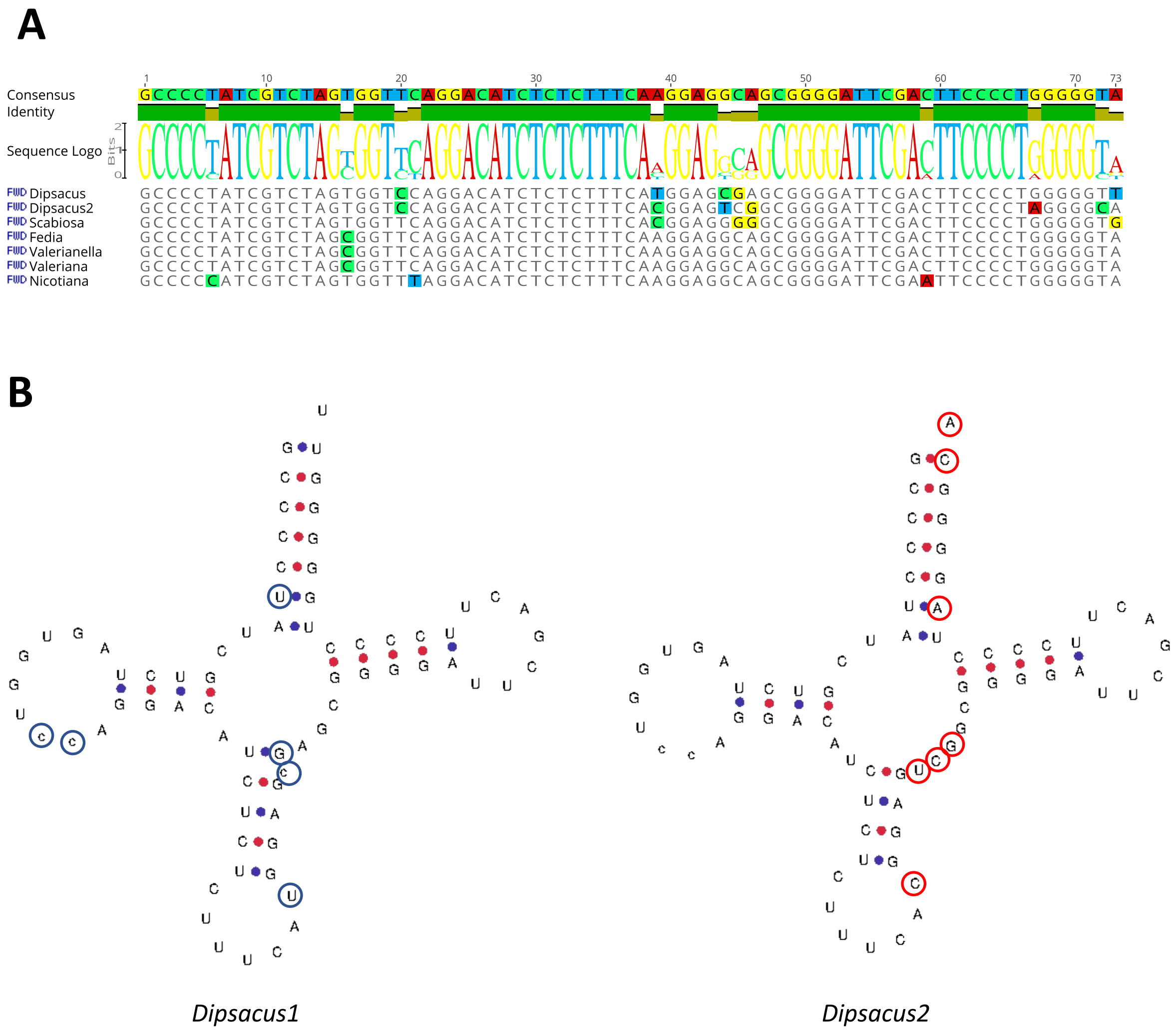

Figure S4. Structural alignments of Caprifoliaceae *s.l.* plastomes using Mauve.

The colored blocks represent collinear sequence blocks shared by all plastomes. Blocks drawn below the horizontal line indicate sequences found in an inverted orientation. Individual genes and strandedness are represented below the *Patrinia* genome block. Only one copy of the inverted repeat (IR) is shown for each plastome and pink boxes below each plastome block indicate its IR. (A) Structural alignments of the subfamilies Dipsacoideae and Valerianoideae. (B) Structural alignments of the subfamily Caprifolioideae and *Patrinia*.

A

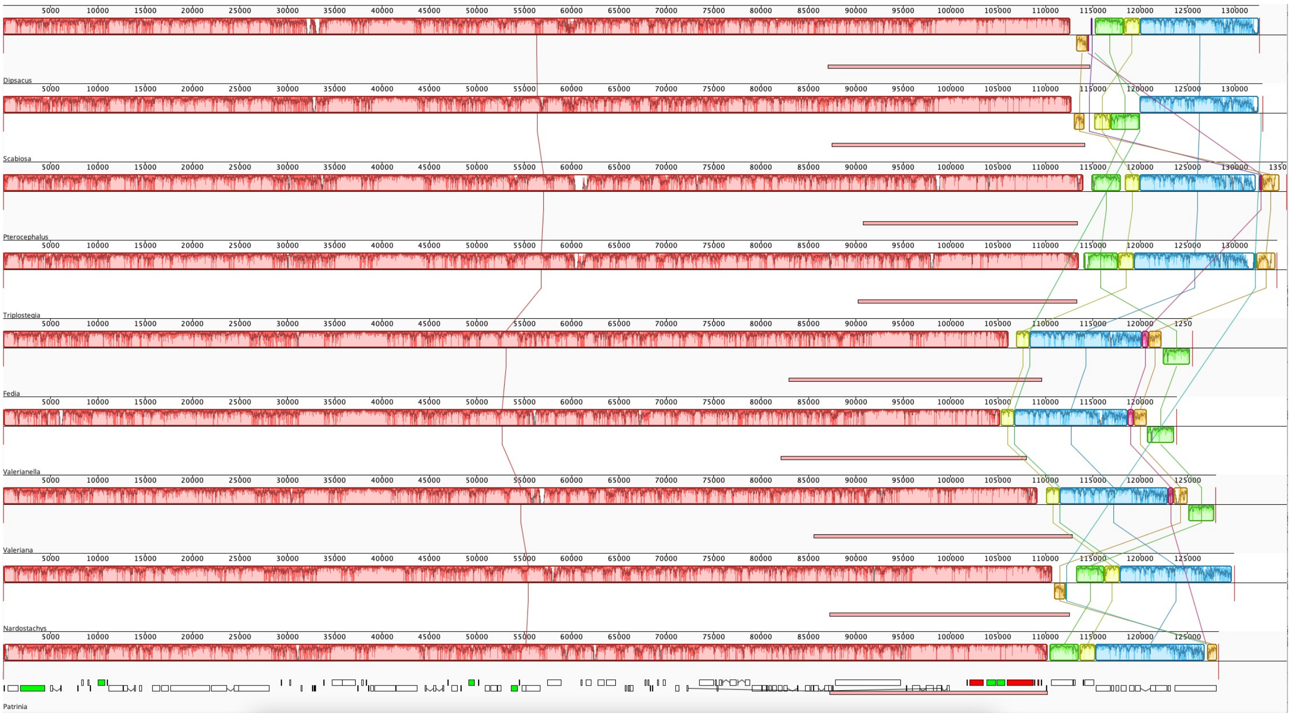

B

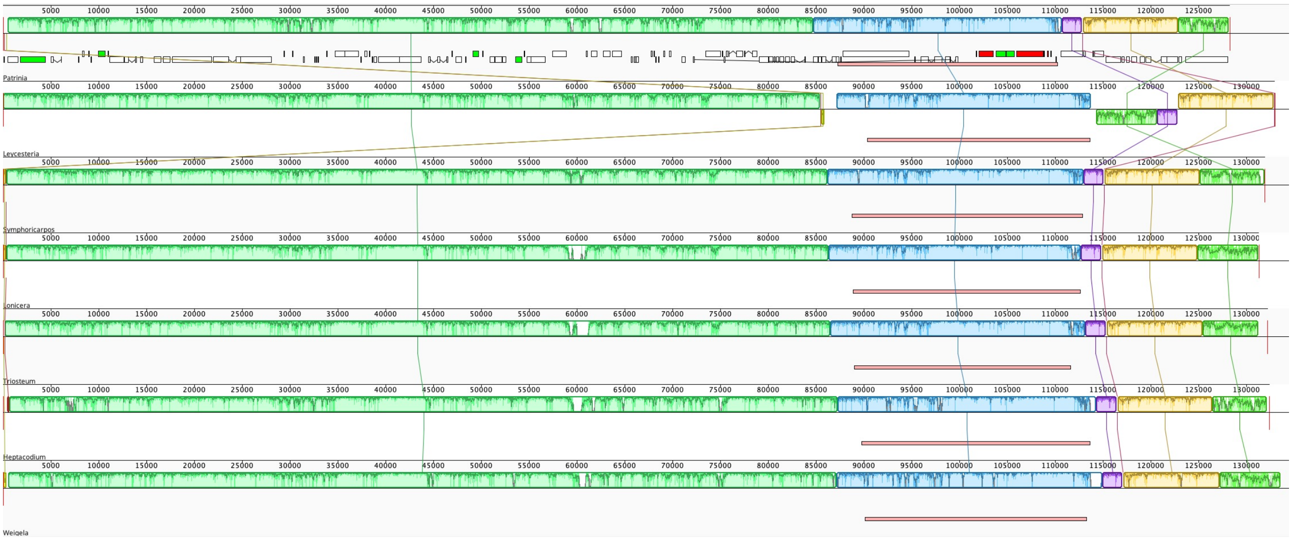

Figure S5. Amino acid sequence alignments of the plastid-encoded *accD* of Caprifoliaceae *s.l.* and outgroups.

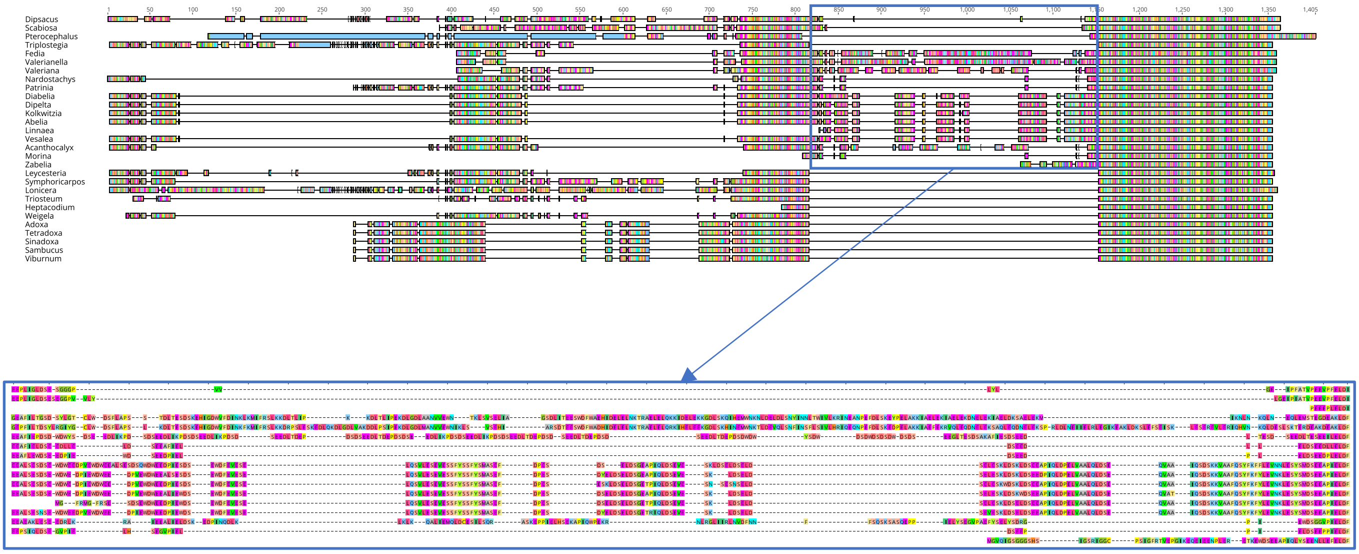

Figure S6. Boxplots of the values of nonsynonymous and synonymous substitution rates of the plastid-encoded *accD* for Caprifoliaceae *s.l.* and outgroups. The box represents values between quartiles, the solid lines extend to the minimum and maximum values, outliers are shown as circles and horizontal lines in the boxes show the median values. Significance of fit was evaluated by Wilcoxon rank sum tests in the R package.

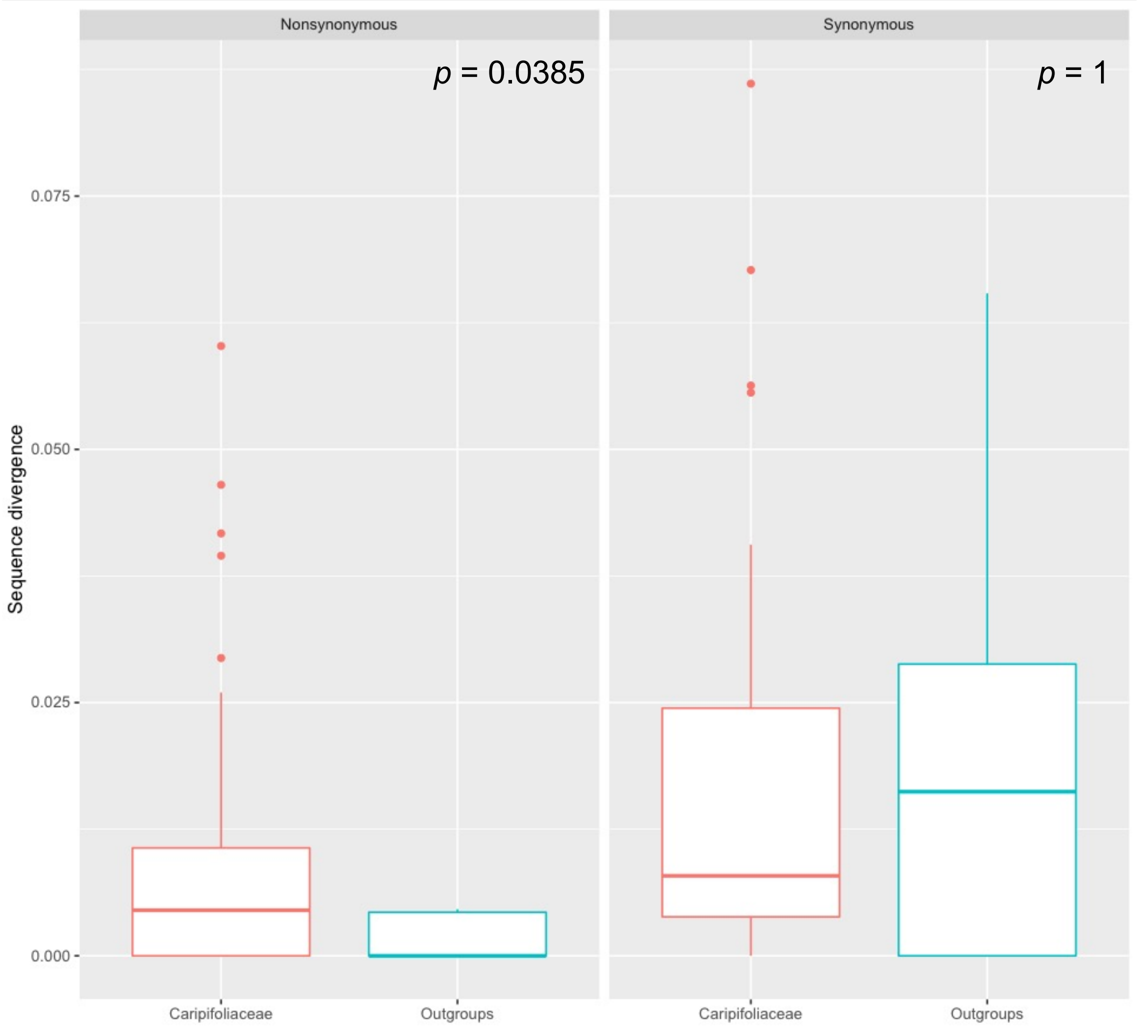

Table S1. GenBank accession numbers for taxa used in this study.

Family	Subfamily	Taxa	NCBI	Repeat pairs	Repeat portion
Caprifoliaceae *s.l.*	Caprifoliaoideae	*Heptacodium miconioides* Rehder	MH712480	67	0.0635
		Leycesteria Formosa Wall.	MN755836	58	0.0367
		Lonicera japonica Thunb.	NC_026839	28	0.0162
		Symphoricarpos orbiculatus Moench	NC_047443	40	0.0239
		Triosteum pinnatifidum Maxim.	NC_037952	52	0.0273
	Dipsacoideae	*Dipsacus japonicus* Miq.	MZ934745	58	0.0316
		Pterocephalus hookeri (C.B.Clarke) E.Pritz.	MN524611	67	0.0624
		Scabiosa comosa Fisch. ex Roem. & Schult.	MZ934747	36	0.0235
		Triplostegia glandulifera Wall. ex DC.	MN524618	71	0.0684
	Divervillideae	*Weigela florida* (Bunge) A.DC.	MN524626	68	0.0586
	Linnaeoldeae	*Abelia macrotera* (Graebn. & Buchw.) Rehder	MN524637	52	0.0603
		Diabelia sanguinea (Makino) Landrein	MK033544	57	0.0598
		Dipelta floribunda Maxim.	MN524641	60	0.0621
		Kolkwitzia amabilis Graebn.	MN524646	62	0.0638
		Linnaea borealis L.	MN524648	60	0.0653
		Vesalea coriacea (Hemsl.) T.Kim & B.Sun ex Landrein	MN524620	72	0.0665
	Monrinoideae	*Acanthocalyx alba* (Hand.-Mazz.) M.J.Cannon	MN524639	74	0.1077
		Morina longifolia Wall. ex DC.	MN524607	95	0.0843
	Valerianoideae	*Fedia cornucopiae* (L.) Gaertn.	MZ934746	24	0.0127
		Nardostachys jatamansi (D.Don) DC.	NC_054306	21	0.0109
		Patrinia saniculifolia Hemsl.	NC_036835	26	0.0128
		Valeriana fauriei Briq.	MZ934748	44	0.019
		Valerianella locusta (L.) Laterr.	MZ934749	11	0.0084
	Zabelioideae	*Zabelia biflora* (Turcz.) Makino ex Hisauti & H.Hara	MN524627	62	0.0568
Adoxaceae	Adoxideae	*Adoxa moschatellina* L.	NC_034792	10	0.0077
		Sambucus williamsii Hance	NC_033878	9	0.0049
		Sinadoxa corydalifolia C.Y.Wu, Z.L.Wu & R.F.Huang	NC_032040	10	0.0072
		Tetradoxa omeiensis (H.Hara) C.Y.Wu	NC_034793	11	0.0078
	Opuloideae	*Viburnum betulifolium* Batalin	NC_037951	14	0.0184

Table S2. Pairwise Wilcoxon rank sum tests of d_N and d_S values among plastid genes within Caprifoliaceae *s.l.*. The Bonferroni correction of d_N and d_S is shown below and above the diagonal, respectively. Bold font indicates significant correlation with $p < 0.05$.

	Abelia	*Acanthocalyx*	*Dipsacus*	*Fedia*	*Scabiosa*	*Valeriana*	*Valerianella*	*Diabelia*	*Dipelta*	*Heptacodium*	*Kolkwitzia*	*Leycesteria*	*Linnaea*	*Lonicera*	*Morina*	*Nardostachys*	*Patrinia*	*Pterocephalus*	*Symphoricarpos*	*Triosteum*	*Triplostegia*	*Vesalea*	*Weigela*	*Zabelia*
Abelia	-	1.0000	1.0000	**0.0871**	1.0000	1.0000	0.1385	1.0000	1.0000	1.0000	1.0000	1.0000	1.0000	1.0000	1.0000	1.0000	1.0000	1.0000	1.0000	1.0000	1.0000	1.0000	1.0000	1.0000
Acanthocalyx	1.0000	-	1.0000	0.4507	1.0000	1.0000	0.6492	1.0000	1.0000	1.0000	1.0000	1.0000	1.0000	1.0000	1.0000	1.0000	1.0000	1.0000	1.0000	1.0000	1.0000	1.0000	1.0000	1.0000
Dipsacus	0.28211	1.0000	-	0.57452	1.0000	1.0000	1.0000	1.0000	1.0000	1.0000	1.0000	1.0000	1.0000	1.0000	1.0000	1.0000	1.0000	1.0000	1.0000	1.0000	1.0000	1.0000	1.0000	1.0000
Fedia	**7.40E-11**	**3.50E-10**	**7.90E-07**	-	1.0000	1.0000	1.0000	0.1131	0.07583	1.0000	**0.0499**	0.05135	0.06387	0.19772	0.31699	1.0000	0.14041	0.92832	**0.01405**	**0.0485**	**0.01541**	0.16692	**0.00044**	**0.00158**
Scabiosa	**0.01842**	0.1255	1.0000	**0.00013**	-	1.0000	1.0000	1.0000	1.0000	1.0000	1.0000	1.0000	1.0000	1.0000	1.0000	1.0000	1.0000	1.0000	1.0000	1.0000	1.0000	1.0000	1.0000	1.0000
Valeriana	**2.00E-07**	**1.40E-06**	**0.00707**	1.0000	0.35826	-	1.0000	1.0000	1.0000	1.0000	1.0000	1.0000	1.0000	1.0000	1.0000	1.0000	1.0000	1.0000	0.4846	1.0000	0.6592	1.0000	**0.0236**	0.0847
Valerianella	**2.10E-11**	**7.70E-11**	**2.70E-07**	1.0000	**5.90E-05**	1.0000	-	0.1719	0.10405	1	0.07701	0.07974	0.09864	0.324	0.43656	1.0000	0.23983	1.0000	**0.02334**	0.0908	**0.02198**	0.25814	**0.00077**	**0.00253**
Diabelia	1.0000	1.0000	0.42003	**2.00E-10**	**0.02637**	**4.70E-07**	**2.70E-11**	-	1.0000	1.0000	1.0000	1.0000	1.0000	1.0000	1.0000	1.0000	1.0000	1.0000	1.0000	1.0000	1.0000	1.0000	1.0000	1.0000
Dipelta	1.0000	1.0000	0.29227	**5.20E-11**	**0.02055**	**2.00E-07**	**1.60E-11**	1.0000	-	1.0000	1.0000	1.0000	1.0000	1.0000	1.0000	1.0000	1.0000	1.0000	1.0000	1.0000	1.0000	1.0000	1.0000	1.0000
Heptacodium	1.0000	1.0000	**0.01143**	**3.20E-11**	**0.00075**	**4.50E-08**	**8.60E-12**	1.0000	1.0000	-	1.0000	1.0000	1.0000	1.0000	1.0000	1.0000	1.0000	1.0000	1.0000	1.0000	1.0000	1.0000	1.0000	1.0000
Kolkwitzia	1.0000	1.0000	0.34355	**6.80E-11**	**0.01905**	**2.40E-07**	**2.30E-11**	1.0000	1.0000	1.0000	-	1.0000	1.0000	1.0000	1.0000	1.0000	1.0000	1.0000	1.0000	1.0000	1.0000	1.0000	1.0000	1.0000
Leycesteria	1.0000	1.0000	0.3735	**9.10E-11**	**0.03345**	**6.10E-07**	**2.90E-11**	1.0000	1.0000	1.0000	1.0000	-	1.0000	1.0000	1.0000	1.0000	1.0000	1.0000	1.0000	1.0000	1.0000	1.0000	1.0000	1.0000
Linnaea	1.0000	1.0000	0.27034	**7.30E-11**	**0.01582**	**2.10E-07**	**2.30E-11**	1.0000	1.0000	1.0000	1.0000	1.0000	-	1.0000	1.0000	1.0000	1.0000	1.0000	1.0000	1.0000	1.0000	1.0000	1.0000	1.0000
Lonicera	1.0000	1.0000	1.0000	**1.20E-10**	0.24289	**1.40E-06**	**2.50E-11**	1.0000	1.0000	1.0000	1.0000	1.0000	1.0000	-	1.0000	1.0000	1.0000	1.0000	1.0000	1.0000	1.0000	1.0000	1.0000	1.0000
Morina	1.0000	1.0000	1.0000	**5.60E-10**	0.45297	**4.20E-06**	**9.80E-11**	1.0000	1.0000	1.0000	1.0000	1.0000	1.0000	1.0000	-	1.0000	1.0000	1.0000	1.0000	1.0000	1.0000	1.0000	1.0000	1.0000
Nardostachys	0.07643	0.46548	1.0000	**5.10E-06**	1.0000	**0.0268**	**1.50E-06**	0.11905	0.06339	**0.00828**	0.10791	0.16163	0.06191	1.0000	1.0000	-	1.0000	1.0000	1.0000	1.0000	1.0000	1.0000	1.0000	1.0000
Patrinia	1.0000	1.0000	1.0000	**4.40E-09**	1.0000	**2.00E-05**	**4.00E-10**	1.0000	1.0000	1.0000	1.0000	1.0000	1.0000	1.0000	1.0000	1.0000	-	1.0000	1.0000	1.0000	1.0000	1.0000	1.0000	1.0000
Pterocephalus	0.0624	0.36073	1.0000	**3.20E-05**	1.0000	0.11907	**8.90E-06**	0.09126	0.05372	**0.00402**	0.06644	0.10156	**0.04768**	0.57022	1.0000	1.0000	1.0000	-	1.0000	1.0000	1.0000	1.0000	1.0000	1.0000
Symphoricarpos	1.0000	1.0000	0.18048	**8.10E-12**	**0.01067**	**6.20E-08**	**2.60E-12**	1.0000	1.0000	1.0000	1.0000	1.0000	1.0000	1.0000	1.0000	0.09267	1.0000	0.03344	-	1.0000	1.0000	1.0000	1.0000	1.0000
Triosteum	1.0000	1.0000	0.24116	**1.80E-11**	**0.01441**	**8.60E-08**	**3.90E-12**	1.0000	1.0000	1.0000	1.0000	1.0000	1.0000	1.0000	1.0000	0.09628	1.0000	0.05633	1.0000	-	1.0000	1.0000	1.0000	1.0000
Triplostegia	1.0000	1.0000	0.72417	**1.30E-10**	0.06095	**6.70E-07**	**2.10E-11**	1.0000	1.0000	1.0000	1.0000	1.0000	1.0000	1.0000	1.0000	0.17785	1.0000	0.12456	1.0000	1.0000	-	1.0000	1.0000	1.0000
Vesalea	1.0000	1.0000	0.24116	**7.80E-11**	**0.02004**	**2.90E-07**	**2.30E-11**	1.0000	1.0000	1.0000	1.0000	1.0000	1.0000	1.0000	1.0000	0.06592	1.0000	0.05458	1.0000	1.0000	1.0000	-	1.0000	1.0000

Weigela	0.58943	0.06489	**3.60E-06**	**1.50E-14**	**1.10E-07**	**1.30E-11**	**4.00E-15**	0.41415	0.48472	1.0000	0.61353	1.0000	0.96343	0.18993	**0.00928**	**2.80E-06**	**0.02837**	**8.90E-07**	1.0000	1.0000	0.33405	0.70065	-	1.0000
Zabelia	1.0000	1.0000	**0.01153**	**1.70E-12**	**0.00055**	**6.40E-09**	**4.10E-13**	1.0000	1.0000	1.0000	1.0000	1.0000	1.0000	1.0000	1.0000	0.00323	1.0000	0.00205	1.0000	1.0000	1.0000	1.0000	1.0000	-

Table S3. Positive selection on Caprifoliaceae *s.l.* plastid genes.

Branch leading to:	gene	d_N/d_S	Holm-Bonferroni corrected *p* value
Dipsacus	*cemA*	1.3354	1.0000
Scabiosa	*cemA*	1.4671	1.0000
	rps4	**1.0755**	**0.0146**
	rps8	1.202	1.0000
Pterocephalus	*matK*	2.5005	1.0000
	rps14	**1.0119**	**0**
Dipsacus/Scabiosa/Pterocephalus	***rps16***	**1.0123**	**0.0000706**
Triplostegia	***ndhF***	**2.8551**	**0.0114**
	rps4	1.9903	1.0000
Fedia	*ndhD*	1.0461	1.0000
Valeriana	***rpoC1***	**1.2861**	**0.0466**
	rps19	1.1748	1.0000
Nardostachys	*ccsA*	1.6913	1.0000
Fedia/Valerianella/Valeriana/Nardostachys	*rpl23*	1.1402	1.0000
	atpF	1.3541	1.0000
Fedia/Valerianella/Valeriana/Nardostachys/Patrinia	*matK*	1.5504	1.0000
	rpl22	1.5492	1.0000
	psbM	1.924	1.0000
Dipsacus/Scabiosa/Pterocephalus/Triplostegia/Fedia/Valerianella/Valeriana/Nardostachys/Patrinia	*atpA*	1.0641	1.0000
Diabelia	*matK*	1.3769	0.8540
Vesalea	***psbM***	**1.2414**	**0.00719**

Abelia	***rps18***	**1.1528**	**0.00241**
Diabelia/Dipelta/Kolkwitzia/Abelia/Linnaea/Vesalea	*infA*	1.557	0.6320
	rpl2	1.9695	1.0000
	rpl14	1.0919	1.0000
	rpoA	1.5454	1.0000
	rpoC1	2.04	1.0000
	rps2	2.0284	1.0000
	rps11	2.8236	1.0000
Acanthocalyx	***infA***	**1.4896**	**0.000207**
	rpl20	1.3174	0.1260
	rps2	**1.261**	**9.96E-08**
	rps11	1.1539	1.0000
	rps18	2.2476	1.0000
	rps19	**1.3798**	**0.0143**
Morina	*atpA*	1.1407	1.0000
	cemA	1.2955	1.0000
	rps8	1.1978	1.0000
	rps11	1.0442	1.0000
	rps19	2.3333	1.0000
Acanthocalyx/Morina	*rpl23*	1.1499	1.0000
	rpl22	2.9095	1.0000
Dipsacus/Scabiosa/Pterocephalus/Triplostegia/Fedia/Valerianella/Valeriana/Nardostachys/Patrinia/Diabelia/Dipelta/Kolkwitzia/Abelia/Linnaea/Vesalea/Acanthocalyx/Morina/Zabelia	***ndhD***	**1.088**	**7.97E-09**
	rpl2	1.6372	1.0000

	rpl20	1.6039	1.0000
Leycesteria	*rpl20*	1.0415	1.0000
	psbJ	1.1546	1.0000
Lonicera	*rps8*	1.2981	1.0000
Triosteum	*atpF*	1.2969	1.0000
Leycesteria/Symphoricarpos/Lonicera/Triosteum	*rpoB*	1.2096	1.0000
	rps7	2.7091	1.0000
Heptacodium	*atpF*	1.1832	1.0000
	rpl32	**1.3509**	**0.000000213**
	rpoC1	2.0817	1.0000
	rps2	2.8455	1.0000
	rps7	2.7203	0.6311
Leycesteria/Symphoricarpos/Lonicera/Triosteum/Heptacodium	*atpB*	1.2396	0.291
Weigela	*atpB*	1.0472	1.0000
	rps4	1.15	1.0000
	rbcL	**1.5877**	**0.0274**
Caprifoliaceae s.l.	*ndhK*	8.1055	1.0000
	rpl16	1.496	1.0000
	rps4	1.6157	1.0000
Adoxa/Tetradoxa/Sinadoxa	***rpl22***	**3.4165**	**0.000209**
Adoxa/Tetradoxa/Sinadoxa/Sambucus	*cemA*	1.5565	1.0000
Viburnum	*rpl33*	1.2695	1.0000
Adoxaceae	*psbC*	1.7453	1.0000

Table S4. CD-search results of the plastid-encoded *accD* gene from Caprifoliaceae *s.l.*.

Family	Subfamily	Query	From	To	E-Value	Bitscore	Domain Accession	Short name
Caprifoliaceae *s.l.*	Dipsacoideae	*Dipsacus*	412	712	0	541.802	CHL00174	accD
			69	284	1.01E-05	48.6307	cl35718	PRK08581 superfamily
			206	409	0.00556986	39.9782	cl35405	PRK05901 superfamily
		Scabiosa	297	597	0	531.016	CHL00174	accD
			196	321	0.000464704	42.6296	cl29916	rplD superfamily
			87	298	0.001719	41.3119	cl35718	PRK08581 superfamily
		Pterocephalus	515	779	0	543.343	CHL00174	accD
		Triplostegia	433	695	0	593.033	CHL00174	accD
	Valerianoideae	*Fedia*	404	605	1.93E-150	435.487	CHL00174	accD
			74	165	1.42E-36	138.498	cl23717	crotonase-like superfamily
			278	409	5.44E-08	55.687	cl41532	pneumo_PspA superfamily
		Valerianella	434	649	3.77E-151	438.954	CHL00174	accD
			60	151	6.20E-36	136.957	cl23717	crotonase-like superfamily
			276	484	2.01E-05	48.0445	cl37666	SMC_N superfamily
			112	405	0	594.189	CHL00174	accD
		Valeriana	147	612	1.88E-168	481.711	CHL00174	accD
			215	405	6.28E-14	75.2494	cl37921	MSCRAMM_SdrC superfamily
		Nardostachys	162	469	0	566.455	CHL00174	accD
		Patrinia	234	541	0	569.921	CHL00174	accD
			154	228	3.83E-05	44.179	cl27567	Apolipoprotein superfamily
	Linnaeoldeae	*Diabelia*	179	629	1.22E-177	505.593	CHL00174	accD
			248	399	1.59E-09	61.1137	cl41468	MSCRAMM_ClfB superfamily

		37	236	0.00191421	41.0102	cl35034	GAT1 superfamily
	Dipelta	179	624	3.21E-178	506.749	CHL00174	accD
		251	394	4.62E-09	59.5729	cl41468	MSCRAMM_ClfB superfamily
	Kolkwitzia	179	626	1.89E-178	507.519	CHL00174	accD
		251	396	6.21E-09	59.1877	cl41468	MSCRAMM_ClfB superfamily
		37	236	0.00324791	40.2398	cl35034	GAT1 superfamily
	Abelia	179	624	7.86E-179	508.29	CHL00174	accD
		248	394	3.29E-08	56.8765	cl41468	MSCRAMM_ClfB superfamily
		37	236	0.00208332	41.0102	cl35034	GAT1 superfamily
	Linnaea	165	365	3.17E-159	448.584	CHL00174	accD
		9	135	5.87E-09	57.6469	cl41468	MSCRAMM_ClfB superfamily
	Vesalea	179	625	1.97E-178	507.519	CHL00174	accD
		248	395	3.40E-07	53.4097	cl41468	MSCRAMM_ClfB superfamily
Monrinoideae	*Acanthocalyx*	151	567	0	524.853	CHL00174	accD
	Morina	48	248	1.84E-166	461.68	CHL00174	accD
Zabelioideae	*Zabelia*	66	267	1.10E-162	452.821	CHL00174	accD
Caprifoliaoideae	*Leycesteria*	206	489	0	586.87	CHL00174	accD
	Symphoricarpos	316	581	0	596.5	CHL00174	accD
		132	307	7.63E-07	52.2541	cl41468	MSCRAMM_ClfB superfamily
	Lonicera	543	824	0	567.995	CHL00174	accD
	Triosteum	1	228	0	514.453	CHL00174	accD
	Heptacodium	1	228	0	519.846	CHL00174	accD
	Weigela	169	456	0	605.36	CHL00174	accD
		23	193	1.94E-07	53.4097	cl41468	MSCRAMM_ClfB superfamily

Adoxaceae	Adoxideae	*Adoxa*	219	505	0	666.992	CHL00174	accD
			49	236	3.11E-05	46.8613	cl41468	MSCRAMM_ClfB superfamily
		Tetradoxa	219	505	0	666.221	CHL00174	accD
			49	236	0.000335689	43.3945	cl41468	MSCRAMM_ClfB superfamily
		Sinadoxa	219	505	0	665.066	CHL00174	accD
			49	236	0.000176245	44.1649	cl41468	MSCRAMM_ClfB superfamily
		Sambucus	219	505	0	665.066	CHL00174	accD
			49	236	0.00310207	40.3129	cl41468	MSCRAMM_ClfB superfamily
	Opuloideae	*Viburnum*	219	505	0	662.755	CHL00174	accD
			49	238	9.25E-05	45.3205	cl41468	MSCRAMM_ClfB superfamily

Table S5. Material information and GenBank accession numbers for length variation in the plastid-encoded *accD* gene.

	Taxon		Information		GenBank accession numbers
for plastome	*Dipsacus japonicus* Miq.		Yeongwol-gun, Gangwon-do, Republic of Korea	20170924	See Table S1
	Scabiosa comosa Fisch. ex Roem. & Schult.		Mt, Palgong, Chilgok-gun, Gyeongsangbuk-do, Republic of Korea	20170930	
	Fedia cornucopiae (L.) Gaertn.		Kew, DNA bank	21556	
	Valeriana fauriei Briq.		Mt. Chaeyak, Yeongcheon-si, Gyeongsangbuk-do, Republic of Korea	20180511	
	Valerianella locusta (L.) Laterr.		Jeongeup-si, Jeollabuk-do, Republic of Korea	20190612	
for *accD* gene	*Valeriana fauriei* Briq.	HB01	Mt. Hambaek, Jeongseon-gun, Gangwon-do, Republic of Korea	20190728	MZ954788
		HB02		20190728	MZ954789
		HB03		20190728	MZ954790
		HB04		20190728	MZ954791
		HB05		20190728	MZ954792
		HB06		20190728	MZ954793
		HB07		20190728	MZ954794
		HB08		20190728	MZ954795
		HB09		20190728	MZ954796
		HB10		20190728	MZ954797
		HB11		20190728	MZ954798
		HB12		20190728	MZ954799
		HB13		20190728	MZ954800
		HB14		20190728	MZ954801
		HB15		20190728	MZ954802
		HB16		20190728	MZ954803
		MS1	Mt, Minjuji, Yeongdong-gun, Chungcheongbuk-do, Republic of Korea	20020524	MZ954804

	MS3		20020524	MZ954805
	MS4		20020524	MZ954806
	MS5		20020524	MZ954807
	MS6		20020524	MZ954808
	MS7		20020524	MZ954809
	MS8		20020524	MZ954810
	MS11		20020524	MZ954811
	SBA1	Mt. Sobaek, Danyang-gun, Chungcheongbuk-do, Republic of Korea	20130613	MZ954812
	SBA8		19970523	MZ954813
	SBA9		19970523	MZ954814
	SBA12		19970523	MZ954815
	SBI2	Mt. Sinbul, Ulju-gun, Ulsan, Republic of Korea	20030517	MZ954816
	SBI3		20080516	MZ954817
	SBI4		20080516	MZ954818
	SBI5		20080516	MZ954819
	SBI6		20080516	MZ954820
	SBI8		20080516	MZ954821
	SBI9		20080516	MZ954822
	SBI10		20080516	MZ954823
	SBI11		20080516	MZ954824
	SBI12		20080516	MZ954825
Valeriana sambucifolia f. dageletiana	UL01	Ulleung-gun, Gyeongsangbuk-do, Republic of Korea	20190703	MZ954826
	UL02		20190703	MZ954827
	UL03		20190703	MZ954828
	UL04		20190703	MZ954829
	UL05		20190703	MZ954830

UL06	20190703	MZ954831
UL07	20190703	MZ954832
UL08	20190704	MZ954833
UL09	20190704	MZ954834
UL10	20190704	MZ954835
UL11	20190704	MZ954836
UL12	20190704	MZ954837
